# Supplementary material for: Comparative analysis of bending moduli in one-component membranes via coarse-grained molecular dynamics simulations
Source: Biophys J. 2025 Jul 16;125(2):432–44. doi: 10.1016/j.bpj.2025.07.014 (PMC12968841; doi:10.1016/j.bpj.2025.07.014)
Supplement: Document S1. Figures S1–S5, Tables S1 and S2, and supporting material [file mmc1.pdf]

Biophysical Journal, Volume *125*

Supplemental information

Comparative analysis of bending moduli in one-component membranes via coarse-grained molecular dynamics simulations

Sam Brown, Jessica Pallarez, and Marat R. Talipov

Supporting Information

Comparative Analysis of the Bending Modulus for Phosphatidylcholine, Phosphatidylethanolamine, and Sphingomyelin Membranes Based on the Coarse-Grained Molecular Dynamics Simulations

Sam Brown, Jessica Pallarez, Marat R. Talipov*

Table of Contents

SI 1. Membrane Generation and Run Protocol 1

SI 2. GROMACS MDP File Parameters 1

SI 3. GROMACS Mean Square Displacement and Diffusion Constant Determination 4

Table S1. Replicability of experiments; Statistics of 5 trials of 100% POPC 4

Figure S1. Quadratic fit of splay to probability density for RSF bending modulus 4

Figure S2. Color coded bending modulus of Thickness vs APL graph 5

Figure S4. Coarse-grained lipids, tail groups and head groups 6

Table S2. Lipid geometrical properties, bending modulus and diffusion from Coarse-Grained Molecular Dynamics Simulations 10

Figure S5. Radial distribution function of POPC and DXPE 12

SI 1. Membrane Generation and Run Protocol.

To generate the membranes for simulations, a python program INSANE was utilized [(https://github.com/Tsjerk/Insane; Wassenaar, et al. 2015]. This program is quite versatile for generation of membranes and membrane systems, allowing many parameters to be defined such as salts, solvent, and embedding proteins into membrane systems. A representative command line for invoking INSANE had the following form: 'python2.7 insane.py -l DNPE:1 -salt 0.15 -x 40 -y 40 -z 20 -pbc cubic -sol W -o system.gro -p system.top -a 0.55'.

Once the membranes were generated, they are then indexed utilizing GROMACS make_ndx interactive command, fully automated with the 'expect' command (https://www.man7.org/linux/man-pages/man1/expect.1.html). After the indexes are generated, the membranes are run through a 2-step preparation process that includes a 10,000-step conjugate gradient minimization and 1,000,000 step NTP equilibration process at 310 K and 1 bar. After equilibration, the systems were subjected to the production step. The parameters used for the minimization, equilibration and production steps can be found in SI 2. The commands used to execute all the molecular dynamics processes (minimization, equilibration and production) are similar, and follow the format: 'gmx grompp -f 0-min.mdp -c system.gro -r system.gro -p system.top -o 00-min.tpr' and 'gmx mdrun -deffnm 00-min -ntomp 24', where grompp produces the .tpr file for the simulation to be run, utilizing the topology and gromacs file produced by INSANE. Finally, the simulation can be started using mdrun with the previously made .tpr file.

SI 2. GROMACS MDP File Parameters

The information below is the parameters used in each .mdp file for GROMACS. 0-min.mdp and 1-md.mdp are the minimization and equilibration for non-cholesterol containing membranes. 2-prod.mdp contains the parameters for the production run.

0-min.mdp:

```
integrator               = steep
nsteps                   = 10000
nstxout                  = 0
nstfout                  = 0
nstlog                   = 100

cutoff-scheme            = Verlet
nstlist                  = 20
pbc                      = xyz
verlet-buffer-tolerance  = 0.005

coulombtype              = reaction-field
rcoulomb                 = 1.1
epsilon_r                = 15    ; 2.5 (with polarizable water)
epsilon_rf               = 0
vdw_type                 = cutoff
vdw-modifier             = Potential-shift-verlet
rvdw                     = 1.1
```

1-md.mdp:

```
; TIMESTEP IN MARTINI
; Most simulations are numerically stable
; with dt=40 fs, some (especially rings and polarizable water) require 20-30 fs.
; Note that time steps of 40 fs and larger may create local heating or
; cooling in your system. Although the use of a heat bath will globally
; remove this effect, it is advised to check consistency of
; your results for somewhat smaller time steps in the range 20-30 fs.
; Time steps exceeding 40 fs should not be used; time steps smaller
; than 20 fs are also not required unless specifically stated in the itp file.

define                   = -DBILAYER_LIPIDHEAD_FC=10
integrator               = md
tinit                    = 0.0
dt                       = 0.020
nsteps                   = 1000000

nstxout                  = 0
nstvout                  = 0
nstfout                  = 0
nstlog                   = 10000 ; Output frequency for energies to log file
nstenergy                = 10000  ; Output frequency for energies to energy file
nstxout-compressed       = 10000  ; Output frequency for .xtc file
compressed-x-precision   = 100

cutoff-scheme            = Verlet
nstlist                  = 50
pbc                      = xyz
verlet-buffer-tolerance  = 0.005

epsilon_r                = 15 ; 2.5 (with polarizable water)
coulombtype              = reaction-field
rcoulomb                 = 1.1
vdw_type                 = cutoff ;(for use with Verlet-pairlist)
rvdw                     = 1.1 ;(for use with Verlet-pairlist)

tcoupl                   = v-rescale
tc-grps                  = MEMB SOLV
```

```
tau_t                    = 1.0  1.0
ref_t                    = 310 310

; Pressure coupling:
Pcoupl                   = C-rescale  ; parrinello-rahman
; Pcoupltype               = isotropic  ; semiisotropic
Pcoupltype               = semiisotropic
tau_p                    = 6.0       ; 12.0  ;parrinello-rahman is more stable with larger tau-p,
DdJ, 20130422
compressibility          = 4.5e-7 4.5e-7
ref_p                    = 1.0  1.0

gen_vel                  = yes
gen_temp                 = 310
gen_seed                 = 473529

constraints              = none
constraint_algorithm     = Lincs
continuation             = no
lincs_order              = 4
lincs_warnangle          = 30

refcoord_scaling         = all
```

2-prod.mdp:

```
integrator               = md
tinit                    = 0.0
dt                       = 0.020
nsteps                   = 50000000

nstxout                  = 50000
nstvout                  = 50000
nstfout                  = 50000
nstlog                   = 50000
nstenergy                = 50000
nstxout-compressed       = 50000
compressed-x-precision   = 100

cutoff-scheme            = Verlet
nstlist                  = 50
pbc                      = xyz
verlet-buffer-tolerance  = 0.005

epsilon_r                = 15
coulombtype              = reaction-field
rcoulomb                 = 1.1
vdw_type                 = cutoff
rvdw                     = 1.1
vdw-modifier             = Potential-shift-verlet

tcoupl                   = v-rescale
tc-grps                  = MEMB SOLV
tau_t                    = 1.0  1.0
ref_t                    = 310 310

; Pressure coupling:
Pcoupl                   = Parrinello-rahman
Pcoupltype               = semiisotropic
tau_p                    = 12.0
compressibility          = 4.5e-5 4.5e-5
ref_p                    = 1.0  1.0

; GENERATE VELOCITIES FOR STARTUP RUN:
```

```
gen_vel                   = no
refcoord_scaling          = all
```

SI 3. GROMACS Mean Square Displacement and Diffusion Constant Determination

To evaluate the lateral diffusion constant, the trajectories were first converted to remove the overall center of mass through use of the following command:

gmx trjconv -f {.xtc file} -n {index file} -s {.tpr file} -center -o {output.xtc}

following which, the mean-square displacement was computed through the next command:

gmx msd -f {.xtc file} -o {output.xvg} -n {index} -lateral z

of which the output file contains the time-labelled mean-square displacement data. This data can then have a linear fit applied to it, the slope of which will be directly proportional to the diffusion constant, by a factor of 2 times the dimensionality (2 for x-y lateral diffusion).

The area per lipid and membrane thickness analysis were performed based on the resulting trajectories using in-house Jupyter Lab python scripts utilizing MDAnalysis.

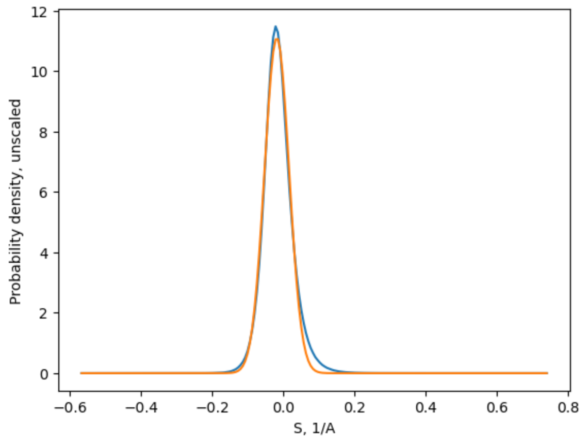

Figure S1. Quadratic fit of splay to probability density for RSF bending modulus.

Table S1. Replicability of experiments; Statistics of 5 trials of 100% POPC (in $k_B T$)

Analysis Method	Mean ± STD
q^{-4}	34.2 ± 0.4 (1.23%)
BW-DCF	24.8 ± 0.3 (1.15%)
RSF	24.3 ± 0.2 (0.719%)

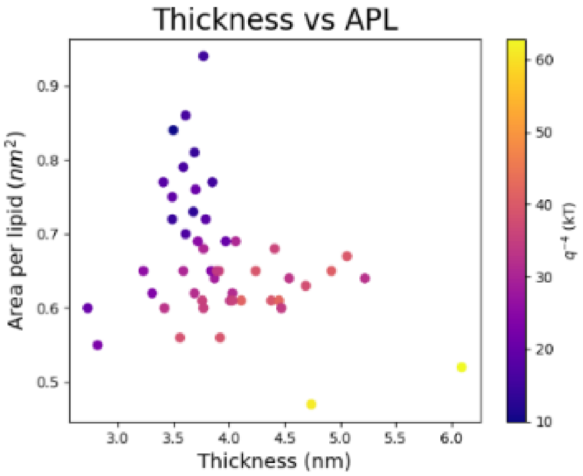

Figure S2. Color coded bending modulus of Thickness vs APL graph.

This plot shows that the high APL values greater than 0.7 nm^2 exclusively have bending moduli at the lower end of the spectrum, and as the APL approaches the more populated section between 0.6 and 0.7 nm^2, the bending modulus is more governed by the thickness of the membrane, but a key relationship has yet to be seen within these geometric parameters of the membranes constituents.

Figure S4. Coarse-grained lipids, tail groups and head groups

This section shows the coarse-grained structures of the lipids ran in simulation for this work.

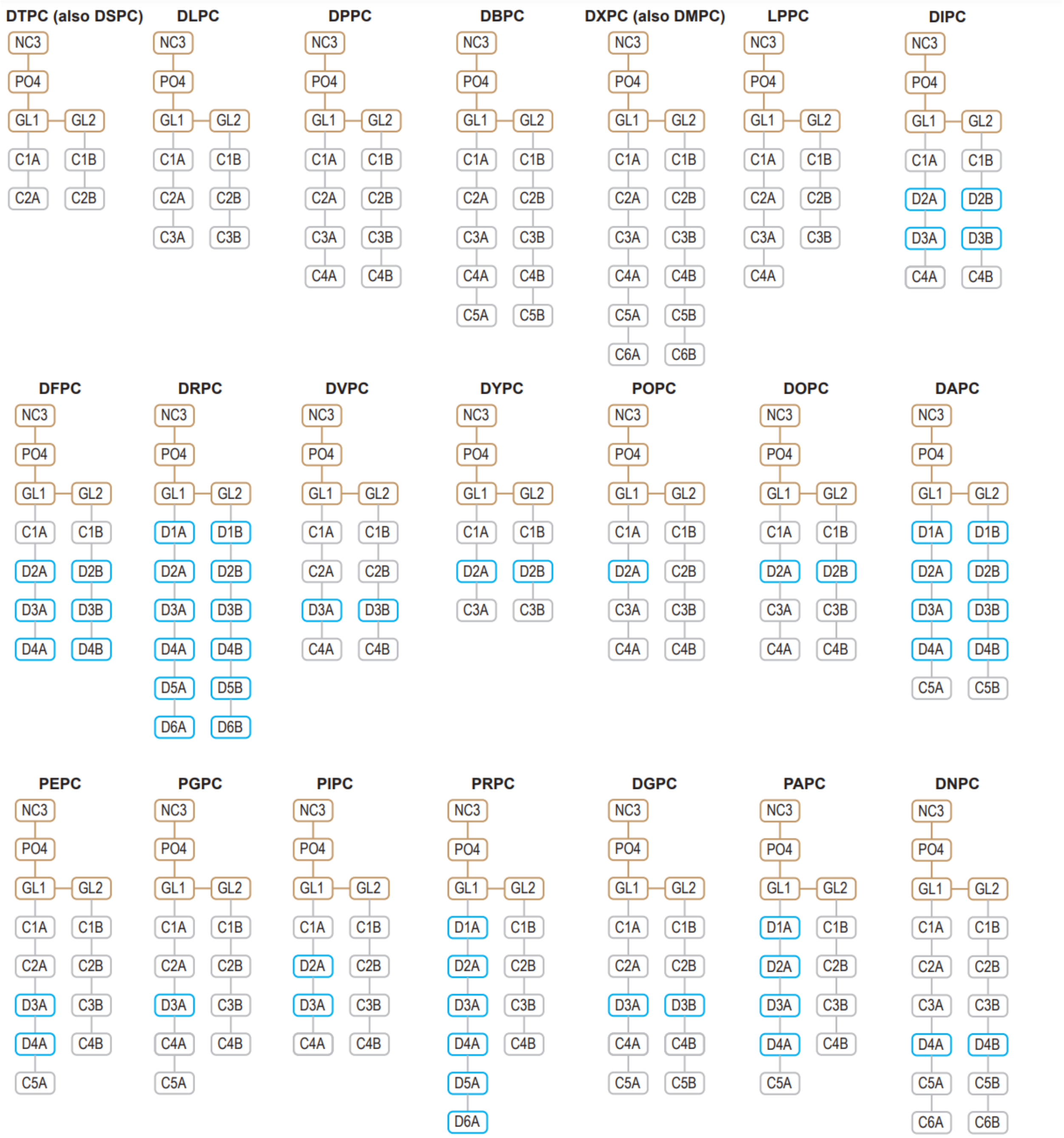

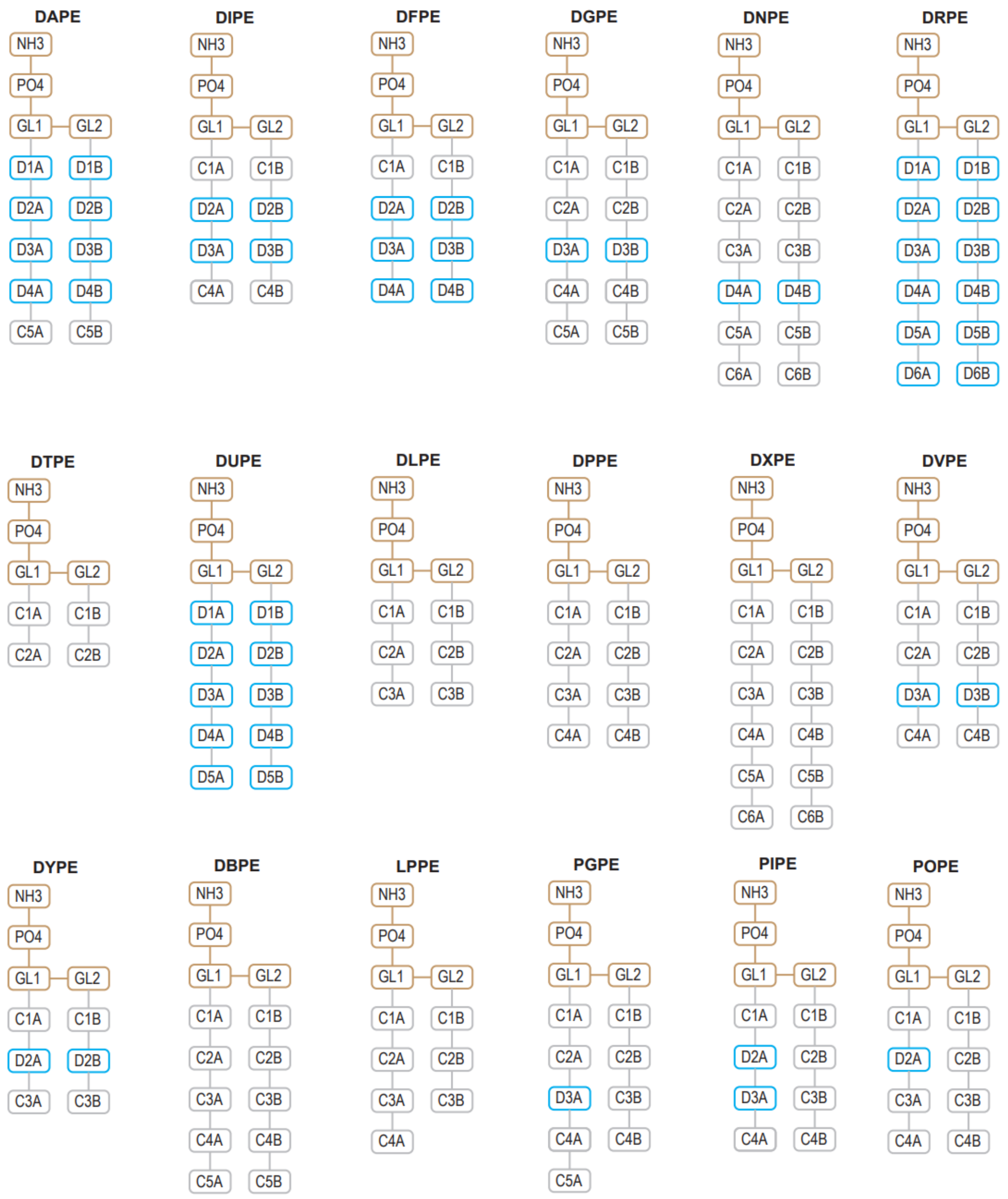
DAPE
NH3 PO4 GL1 GL2 D1A D1B D2A D2B D3A D3B D4A D4B C5A C5B
DIPE
NH3 PO4 GL1 GL2 C1A C1B D2A D2B D3A D3B C4A C4B
DFPE
NH3 PO4 GL1 GL2 C1A C1B D2A D2B D3A D3B D4A D4B
DGPE
NH3 PO4 GL1 GL2 C1A C1B C2A C2B D3A D3B C4A C4B C5A C5B
DNPE
NH3 PO4 GL1 GL2 C1A C1B C2A C2B C3A C3B D4A D4B C5A C5B C6A C6B
DRPE
NH3 PO4 GL1 GL2 D1A D1B D2A D2B D3A D3B D4A D4B D5A D5B D6A D6B
DTPE
NH3 PO4 GL1 GL2 C1A C1B C2A C2B
DUPE
NH3 PO4 GL1 GL2 D1A D1B D2A D2B D3A D3B D4A D4B D5A D5B
DLPE
NH3 PO4 GL1 GL2 C1A C1B C2A C2B C3A C3B
DPPE
NH3 PO4 GL1 GL2 C1A C1B C2A C2B C3A C3B C4A C4B
DXPE
NH3 PO4 GL1 GL2 C1A C1B C2A C2B C3A C3B C4A C4B C5A C5B C6A C6B
DVPE
NH3 PO4 GL1 GL2 C1A C1B C2A C2B D3A D3B C4A C4B
DYPE
NH3 PO4 GL1 GL2 C1A C1B D2A D2B C3A C3B
DBPE
NH3 PO4 GL1 GL2 C1A C1B C2A C2B C3A C3B C4A C4B C5A C5B
LPPE
NH3 PO4 GL1 GL2 C1A C1B C2A C2B C3A C3B C4A
PGPE
NH3 PO4 GL1 GL2 C1A C1B C2A C2B D3A C3B C4A C4B C5A
PIPE
NH3 PO4 GL1 GL2 C1A C1B D2A C2B D3A C3B C4A C4B
POPE
NH3 PO4 GL1 GL2 C1A C1B D2A C2B C3A C3B C4A C4B

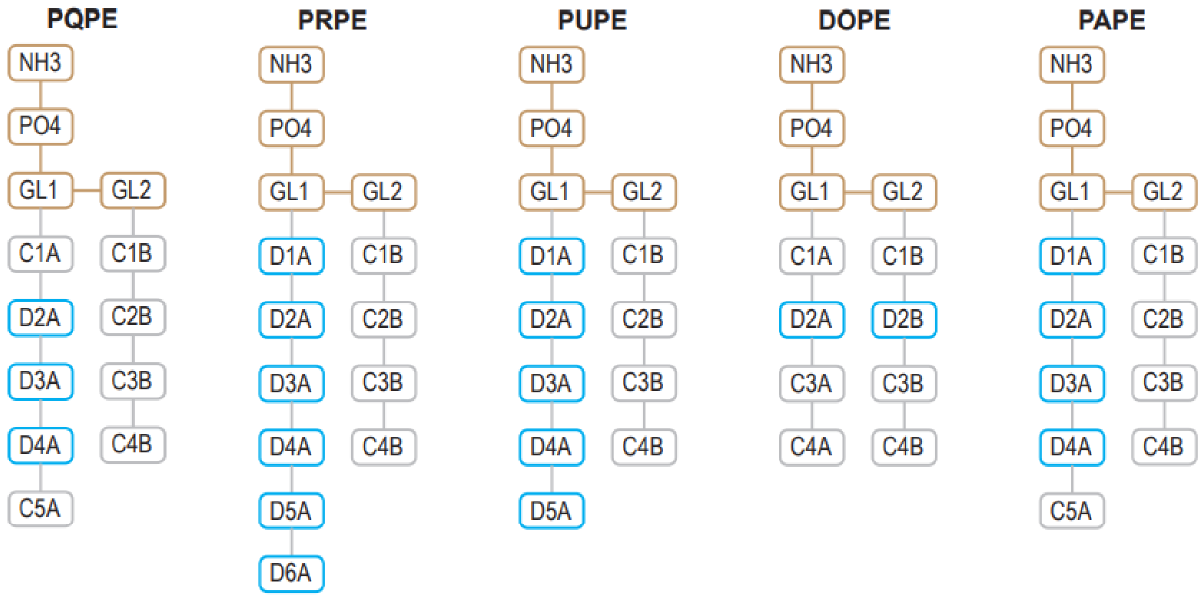
PQPE
NH3
PO4
GL1
GL2
C1A
D2A
D3A
D4A
C5A
C1B
C2B
C3B
C4B
PRPE
NH3
PO4
GL1
GL2
D1A
D2A
D3A
D4A
D5A
D6A
C1B
C2B
C3B
C4B
PUPE
NH3
PO4
GL1
GL2
D1A
D2A
D3A
D4A
D5A
C1B
C2B
C3B
C4B
DOPE
NH3
PO4
GL1
GL2
C1A
D2A
C3A
C4A
C1B
D2B
C3B
C4B
PAPE
NH3
PO4
GL1
GL2
D1A
D2A
D3A
D4A
C5A
C1B
C2B
C3B
C4B

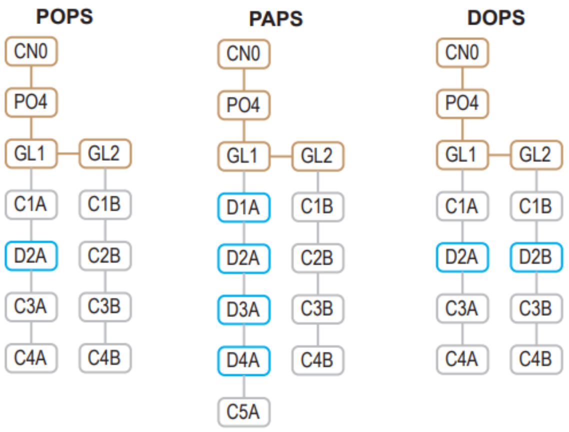

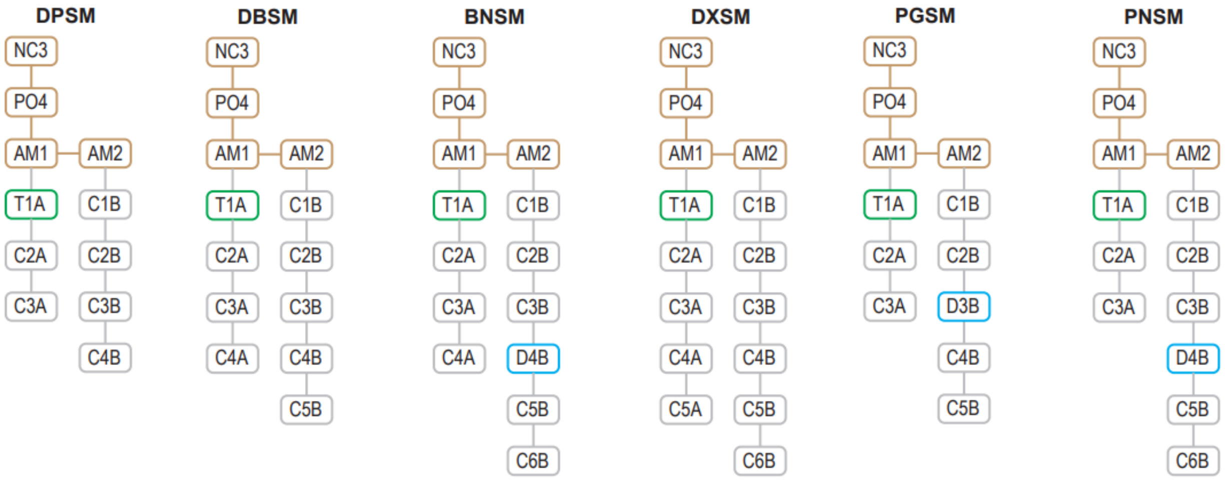

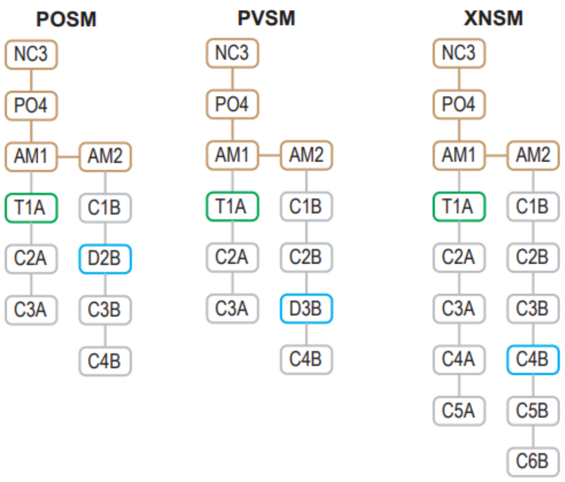

In the four-letter nomenclature of membranes used in this study, a T1A bead is equivalent to two C-type units. For example, in SM-type lipids, the abbreviation P represents a -TCC tail, whereas in PC, PE, and PS lipids, P denotes a -CCCC tail.

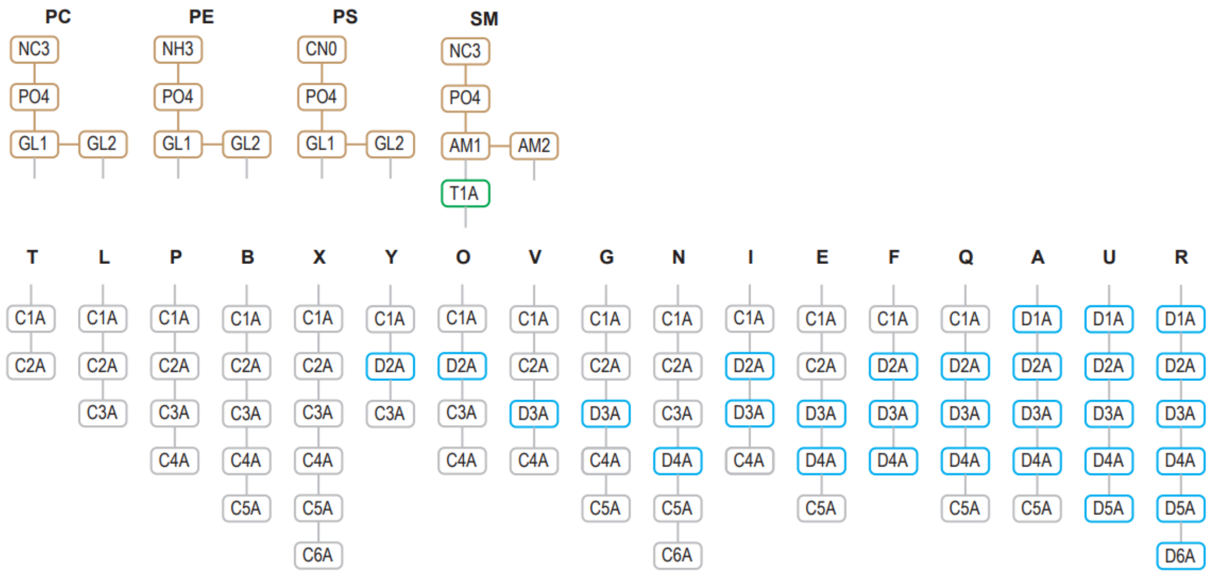

Table S2. Lipid geometrical properties, bending modulus and diffusion from Coarse-Grained Molecular Dynamics Simulations

Lipid type	APL (nm^2)	D_B (nm)	$\kappa(q^{-4})(k_BT)$	κ(BW-DCF) (k_BT)	κ(RSF) (k_BT)	D (10^{-6} cm^2/s)
100% DTPC	0.6	2.73	20.11	16.16	30.68	1.705
100% DLPC	0.6	3.42	32.8	24.25	31.02	1.079
100% DGPC	0.68	4.41	36.03	27.99	21.86	0.5956
100% DNPC	0.67	5.06	39.68	36.97	21.49	0.3503
100% PEPC	0.69	4.06	30.44	20.31	21.66	0.7977
100% DYPC	0.65	3.23	25.79	18.22	25.32	1.165
100% DVPC	0.65	3.91	36.57	25.8	24.78	0.7237
100% DFPC	0.77	3.41	17.81	12.48	18.08	1.489
100% LPPC	0.61	3.76	35.55	28.35	29.82	0.9979
100% PGPC	0.65	4.24	39.1	30.99	24.66	0.6036
100% DAPC	0.86	3.61	15.89	10.16	13.36	1.495
100% DTPE	0.55	2.82	22.91	15.69	40.58	1.308
100% DLPE	0.56	3.56	38.34	23.5	41.88	0.913
100% DOPE	0.64	3.87	28.7	14.48	26.12	0.7498
100% DIPE	0.7	3.61	16.56	9.47	20.2	1.251
100% DFPE	0.72	3.49	14.17	8.84	20.06	1.255
100% DGPE	0.64	4.54	33.16	19.36	25.51	0.4402
100% DAPE	0.81	3.69	13.72	7.13	15.78	1.41
100% DNPE	0.64	5.22	32.33	23.77	25.82	0.4245
100% PGPE	0.61	4.38	39.22	23.22	29.99	0.5107
100% PQPE	0.69	3.97	20.2	11.02	22.34	0.8613
100% DBSM	0.61	4.45	42.07	41.11	29.75	0.5308

100% PVSM	0.62	3.69	31.8	27.97	28.01	0.7873
100% BNSM	0.63	4.69	37.69	38.71	23.56	0.5073
100% PGSM	0.62	4.03	31.37	26.29	24.31	0.6973
100% PNSM	0.6	4.47	34.25	31.28	23.09	0.5302
100% POSM	0.65	3.59	30.21	24.44	25.03	0.8901
100% XNSM	0.65	4.92	40.72	41.22	24.87	0.4179
100% DIPC	0.75	3.49	19.05	12.99	17.96	1.292
100% DOPC	0.68	3.77	30.47	21.36	21.99	1.0919
100% DPPC	0.61	4.10	41.23	34.66	30.14	0.7735
100% DPSM	0.6	3.77	35.05	30.39	29.44	0.7399
100% DVPE	0.61	4.01	33.34	18.04	29.44	0.6407
100% DXPE	0.46	6.5	58.2	135.47	135.87	0.01084
100% DYPE	0.62	3.31	23.74	13.57	29.88	1.0468
100% LPPE	0.56	3.92	38.9	24.15	40.1	0.7206
100% PAPC	0.76	3.7	20.31	13.31	17.43	1.2687
100% PAPE	0.72	3.79	18.2	9.58	18.42	1.0177
100% PIPC	0.69	3.72	25.14	16.96	21.31	0.9053
100% PIPE	0.65	3.84	24.33	12.31	25.06	0.8508
100% POPC	0.65	3.89	34.27	25.84	24.57	0.7326
100% POPE	0.61	4.03	35.17	19.10	30.14	0.6933
100% PUPE	0.74	3.68	13.27	7.38	13.96	1.0165
100% DRPC	0.94	3.77	15.06	8.66	12.74	1.3079
100% DUPE	0.84	3.5	9.91	4.74	14.43	1.2569
100% PRPE	0.77	3.85	15.24	6.22	16.44	0.7569
100% PUPC	0.79	3.59	16.03	10.57	14.92	1.1821
100% DBPC	0.61	4.78	40.48	45.62	29.97	0.4883
100% DPPE	0.54	4.41	47.88	46.3	55.41	0.3708
100% DXPC	0.62	5.45	37.97	51.81	29.68	0.3381
100% DXPE	0.46	6.47	70.22	84.44	142.7	0.02195
100% DXSM	0.61	5.12	37.76	52.64	29.57	0.3488

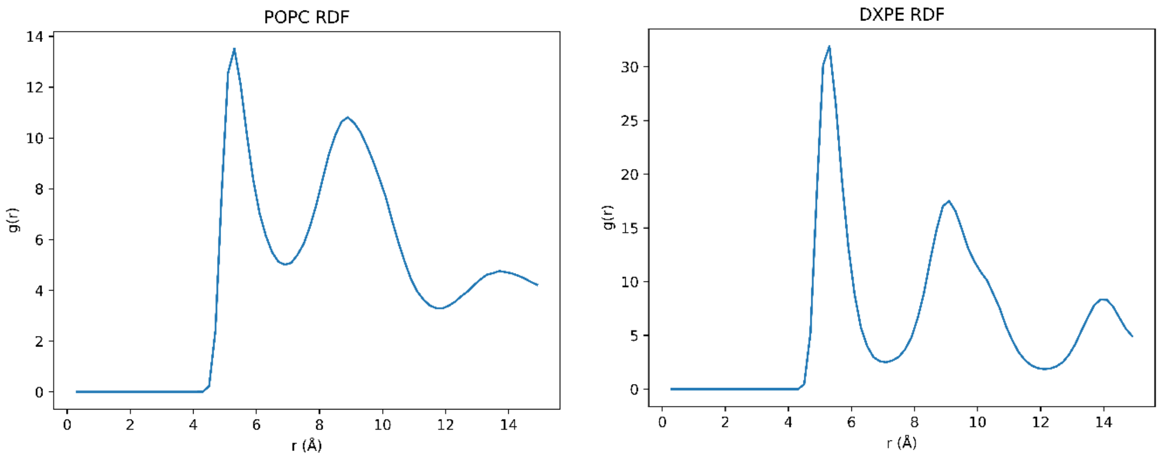

Figure S5. Radial distribution function of POPC and DXPE membranes. DXPE sharpened peaks indicate a more ordered (gel) phase.
