# Supplementary material for: Comparative analysis of bending moduli in one-component membranes via coarse-grained molecular dynamics simulations
Source: Biophys J. 2025 Jul 16;125(2):432–44. doi: 10.1016/j.bpj.2025.07.014 (PMC12968841; doi:10.1016/j.bpj.2025.07.014)
Supplement: Document S2. Article plus supporting material [file mmc2.pdf]

Biophysical Journal
Article

Biophysical Society

Comparative analysis of bending moduli in one-component membranes via coarse-grained molecular dynamics simulations

Sam Brown,[1] Jessica Pallarez,[1] and Marat R. Talipov[1,*]
[1]Department of Chemistry and Biochemistry, New Mexico State University, Las Cruces, New Mexico

ABSTRACT Bilayer membranes are essential biological structures with complex and largely unexplored mechanical properties. Using coarse-grained molecular dynamics simulations, we evaluated the bending modulus across diverse lipid compositions, including phosphatidylcholine, phosphatidylethanolamine, and sphingomyelin. Three computational techniques were employed to calculate the bending modulus from thermal fluctuations of the simulated bilayers: the Fourier transform of the lipid height function (q^{-4} fitting), the Bedeaux-Weeks density correlation function method, and real space fluctuations. The analysis revealed substantial variations in bending modulus values across methods, underscoring the inherent complexities and discrepancies in computational assessments. These findings advance our understanding of membrane dynamics and provide valuable insights into bilayer structural behavior. The results support the broader application of computational approaches to study biological systems and inspire the development of biomimetic materials with tailored mechanical properties.

SIGNIFICANCE The mechanical properties of lipid membranes play a critical role in various biological and technological applications, yet a systematic comparative analysis of their bending moduli remains underexplored. This study addresses this gap by providing computational insights into the bending stiffness of biologically relevant lipid membranes. Our results offer valuable implications for biophysical research, drug delivery systems, and biomembrane engineering.

INTRODUCTION

Bilayer membranes play a crucial role in cellular systems, acting as a boundary between distinct environments and as dynamic platforms for biological interactions (1). Mechanical properties of membranes are crucial for the membrane's function and adaptability in biological systems, including vesicle formation, membrane fusion, and the morphology of organelles (2). One of these properties, the bending modulus, is indicative of the energy required to deform a membrane, where a higher modulus suggests greater rigidity (3). Quantification of the bending modulus can provide insights into more complex biological systems, such as transmembrane protein systems and enclosed cellular systems. As an example, a mechanosensitive transmembrane ion channel protein complex, Piezo1, has been stated to change conformations when the membrane around the protein undergoes a localized flattening associated with the local concentration changes (4).

Several experimental techniques have been used to indirectly measure the bending modulus of cell membranes, including micropipette aspiration and x-ray scattering (5). Experimental determination of the bending modulus is challenging, often yielding variable results across techniques (5), as shown in Table 1. The table shows that the estimated bending modulus values can vary quite drastically depending on the method used, as well as show variability for the duplicated runs and unexpected trends, such as an increase of rigidity with increased temperature. These studies, although foundational, do not fully capture the complexity and variability of the membrane composition observed *in vivo*. In light of these challenges, computational studies provide a valuable complementary technique for exploring the bending moduli of membranes through the analysis of the membrane dynamics and its thermal fluctuations (6–11). Such studies enable systematic variation of lipid

Submitted March 13, 2025, and accepted for publication July 11, 2025.
*Correspondence: talipovm@nmsu.edu
Editor: Ragothaman M. Yennamalli.
https://doi.org/10.1016/j.bpj.2025.07.014
© 2025 The Authors. Published by Elsevier Inc. on behalf of Biophysical Society.
This is an open access article under the CC BY license (http://creativecommons.org/licenses/by/4.0/).

TABLE 1 Experimentally determined bending modulus values for 100% POPC membranes [a]

Experimental technique	T (K)	κ_b (k_BT)
Electro-deformation	297.15	14.14
Fluctuation analysis	297.15	9.51; 35.6[b]
X-ray scattering	303.15	20.32
Micropipette aspiration	298.15	51.28
Neutron spin echo and dynamic light scattering	295.15	18.9

[a]Fluctuation analysis varies greatly, even at the same temperature (5).

types without altering other physical parameters, providing a controlled environment, decoupled from other biological variables, to study the effects of composition on membrane properties.

This paper compares three computational approaches for determining the bending modulus (κ), building on the foundational work of Helfrich (3). Helfrich's model describes the membrane as a plane with an associated height, establishing a geometric framework for its analysis. The approaches include fluctuation spectrum analysis, as detailed, e.g., by Brandt et al. (11); a method developed by Khelashvili et al. (6–10), which focuses on lipid neighbor pairs and their tilt angles; and a technique introduced by Hernández-Muñoz et al. (12,13), which applies density correlation functions integrated into capillary wave theory. By comparing these methods, we analyze bending modulus values across various membrane compositions, providing enhanced insights into membrane dynamics in biological systems.

MATERIALS AND METHODS

Computational details

The membrane bilayers involved in this study are composed of amphiphilic lipids, each consisting of a hydrophilic head and two hydrophobic tails. Some examples are shown in Fig. 1, featuring the all-atom (AA) and coarse-grained (CG) representation of selected groups, with a full set of CG tail groups and lipids, together with the lipid name abbreviations, shown in Fig. S4. The CG representation, based on the Martini 2.2 force field, simplifies the molecular structure by replacing groups of atoms with single centroid beads.

To examine and quantify the bending modulus of membranes, we employed a molecular dynamics (MD) approach using the CG Martini 2.2 force field (14). We used a nonbonded interaction cutoff distance of 1.1 nm for the Coulomb and the Lennard-Jones potentials.

CG force fields, such as Martini, produce smoother potential energy surfaces compared to the AA force fields, which allows the use of larger time steps. In this paper, the simulations were run for 1 μs using 20 fs time steps at 310 K and 1 atm, with a simulation box of 40 × 40 × 20 nm. The simulations were performed using GPU-enabled GROMACS 2023.1 software (15).

The initial structural configurations of membranes within the solvent box were generated using the Python program INSANE, as described by Wassenaar et al. (16). The generated configurations were subjected to a two-step preparation process that includes a 10,000-step conjugate gradient minimization, followed by a 1,000,000 step NPT equilibration at 310 K and 1 bar. The coordinates and velocities from the final equilibration frame were used to initialize the 1 μs-long production simulation. Further details about the workflow can be found in section 1 of the supporting material, and the molecular dynamics parameters (MDP) files can be found in section 2 of the supporting material.

The lateral diffusion constants were calculated from trajectories with the overall center of mass motion removed. The lateral mean-squared displacement (MSD) data were obtained through GROMACS utilizing its built-in MSD command (see section 3 of the supporting material). The lateral MSD data were fitted to a linear model, where the slope is directly proportional to the diffusion constant by a factor of four, arising from two times the dimensionality of the diffusion (i.e., $D = 2$).

The area per lipid (APL) and membrane thickness were analyzed using the resulting trajectories using in-house Python scripts (17) developed in JupyterLab Notebook (https://jupyter.org) using the MDAnalysis package (18,19). The bending modulus values from the production run trajectories were analyzed using the techniques discussed in the next section, also implemented using MDAnalysis.

Additional computational details, together with the command-line scripts, are provided in the supporting material.

Theoretical details

This section details three methods used to determine the bending modulus of membranes from simulations. These three methods are referred to as the q^{-4} method (based on the Fourier transform of the lipid height function) (3,11,20), the Bedeaux-Weeks density correlation function (BW-DCF) (12,13), and the real space fluctuation (RSF) method (6–10).

The first method, the q^{-4} approach, is based on the Helfrich Hamiltonian, which describes the relationship between the force and curvature in a membrane. By taking an area integral, the Hamiltonian provides a relation between energy and curvature, as shown in Eq. 1:

$$E = \int \left(\tau + \frac{\kappa_b}{2} K^2 \right) dA. \tag{1}$$

Here, τ represents the membrane tension, κ_b is the bending modulus, and K is the total curvature of the system. The total curvature can be represented as $K = C_1 + C_2$, where C_1 and C_2 denote the directional curvatures.

The following derivations, which form the basis of this analysis method, are drawn from the work of Brandt (11) and Boal (20). Vectors are denoted in bold font (e.g., $\mathbf{q}$), whereas their magnitudes appear without bold (e.g., q).

The membrane surface $\mathbf{r}$ is modeled as an x-y plane with an associated height that is a function of x and y, i.e., $\mathbf{r} = [x, y, h(x, y)]$. The actual area of the membrane can be represented as

$$dA = \frac{\partial \mathbf{r}}{\partial x} dx \times \frac{\partial \mathbf{r}}{\partial y} dy = \left| \partial_x \mathbf{r} \times \partial_y \mathbf{r} \right| dx\, dy. \tag{2}$$

The partial derivatives of $\mathbf{r}$ with respect to x and y are given by

$$\partial_x \mathbf{r} = [1, 0, \partial_x h] = [1, 0, h_x] \text{ and} \tag{3a}$$

$$\partial_y \mathbf{r} = \left[0, 1, \partial_y h\right] = \left[0, 1, h_y\right]. \tag{3b}$$

Substituting these expressions into the equation for dA and taking the norm of the cross product yields

$$dA = \left(h_x^2 + h_y^2 + 1 \right)^{\frac{1}{2}} dx\, dy. \tag{4}$$

The term under the square root in dA is referred to as the metric, g. To further analyze the membrane, we now represent the curvature of the

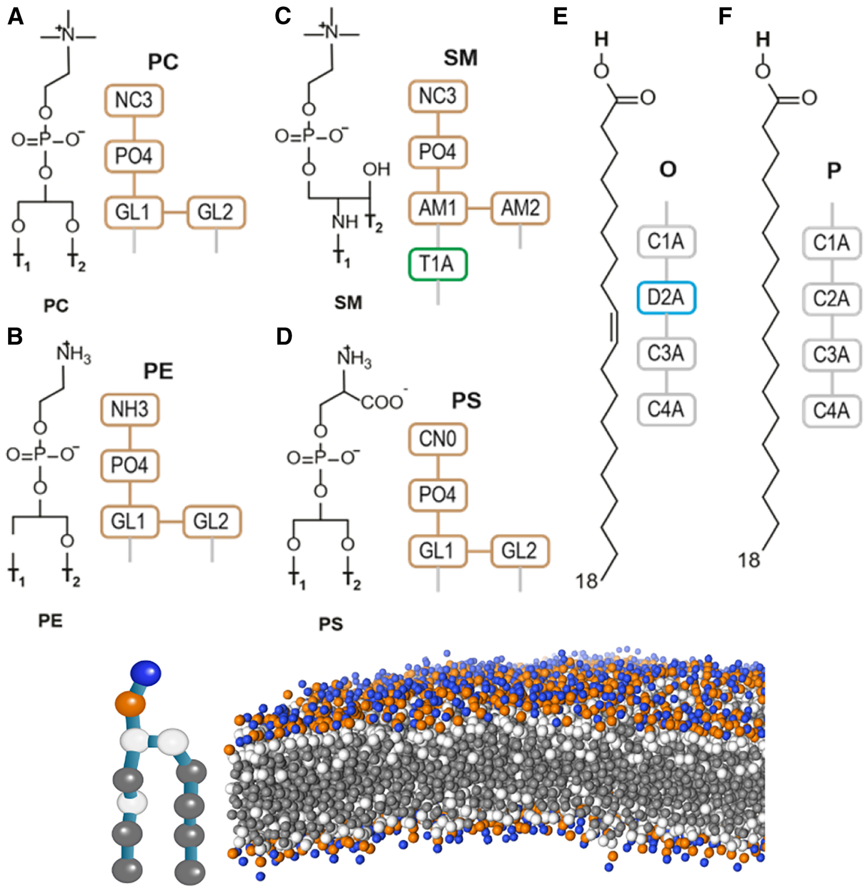

FIGURE 1 Structures of common phospholipid headgroups: (*A*) phosphatidylcholine (PC), (*B*) phosphatidylethanolamine (PE), (*C*) sphingomyelin (SM), and (*D*) phosphatidylserine (PS). (*E*) and (*F*) show coarse-grained representations of the oleoyl and palmitoyl tail groups.

membrane using the height function. From differential geometry, the curvature of a surface may be expressed as

$$C = \boldsymbol{n} \cdot \left(\frac{\partial^2 \boldsymbol{r}}{\partial s^2} \right). \tag{5}$$

Here, $\boldsymbol{n}$ represents the unit normal vector and s denotes the arc length along the surface. Differentiating $\boldsymbol{r}$ with respect to s yields

$$\frac{\partial^2 \boldsymbol{r}}{\partial s^2} = \boldsymbol{r}_{xx} \left(\frac{\partial x}{\partial s} \right)^2 + \boldsymbol{r}_x \left(\frac{\partial^2 x}{\partial s^2} \right) + \boldsymbol{r}_{yy} \left(\frac{\partial y}{\partial s} \right)^2 + \boldsymbol{r}_y \left(\frac{\partial^2 y}{\partial s^2} \right) + 2\boldsymbol{r}_{xy} \frac{\partial x}{\partial s} \frac{\partial y}{\partial s} \quad . \tag{6}$$

The second and fourth terms in this derivative do not contribute to the orthogonality $\boldsymbol{n} \cdot \boldsymbol{r}_\alpha = 0$ and hence can be dropped out. The remaining second-derivative terms are combined with $\boldsymbol{n}$ to represent a coefficient, b, where $b_{\alpha\beta} = \boldsymbol{n} \cdot \boldsymbol{r}_{\alpha\beta}$. Differentiating the orthogonality condition of $\boldsymbol{n} \cdot \boldsymbol{r}_\alpha = 0$ with respect to β and combining it with the definition of b yields $b_{\alpha\beta} = -\boldsymbol{n}_\beta \cdot \boldsymbol{r}_\alpha$. These coefficients b can then be combined with the determinant of the previously defined metric tensor g, where $g_{\alpha\beta} = \boldsymbol{r}_\alpha \cdot \boldsymbol{r}_\beta$, into Eq. 7a, which is derived from Eq. 5 and represents the mean curvature:

$$\frac{(C_1 + C_2)}{2} = \frac{g_{xx} b_{yy} + g_{yy} b_{xx} - 2 g_{xy} b_{xy}}{2g}. \tag{7a}$$

Calculating and substituting in the identities of partial derivatives of g and b simplifies Eq. 7a down to Eq. 7b:

$$\frac{C_1 + C_2}{2} = \frac{1}{2g^{\frac{3}{2}}} \left[h_{yy} \left(h_x^2 + 1 \right) + h_{xx} \left(h_y^2 + 1 \right) - 2 h_x h_y h_{xy} \right]. \tag{7b}$$

For slow undulations, this mean curvature expression can be approximated as

$$\frac{C_1 + C_2}{2} \cong \frac{h_{xx} + h_{yy}}{2}. \tag{7c}$$

We can now implement Eqs. 4 and 7c to define the Helfrich Hamiltonian as

$$E = \frac{1}{2} \int \left\{ \tau \left(h_x^2 + h_y^2 \right) + \kappa_b \left(h_{xx} + h_{yy} \right)^2 \right\} d\boldsymbol{X}, \tag{8}$$

where $d\boldsymbol{X} = dx\, dy$.

The Helfrich Hamiltonian can be inverse Fourier transformed into the frequency domain. The height function can be represented as

$$h(\boldsymbol{X}) = \frac{A}{4\pi^2} \int e^{i\boldsymbol{q} \cdot \boldsymbol{X}} h(\boldsymbol{q}) d\boldsymbol{q}, \tag{9a}$$

where A is the projected area. Fig. 2 shows a representation of the Fourier transform process.

Differentiating and taking the complex square of this inverse Fourier transform shows

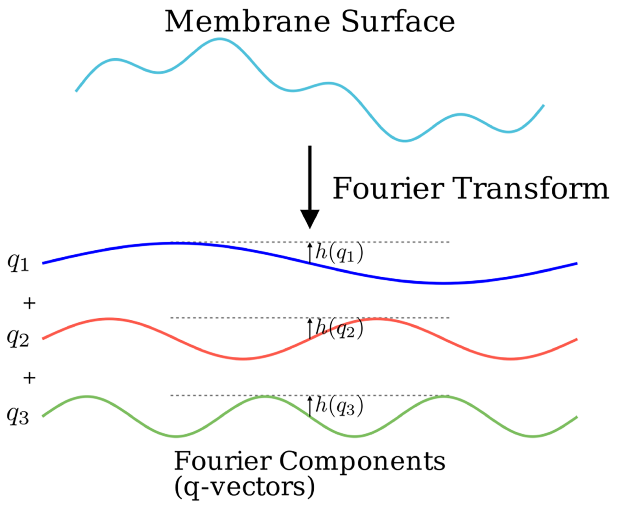

FIGURE 2 Visual representation of the Fourier transform process, transforming from spatial to frequency domain

$$h_x(\boldsymbol{X})^2 = \left[\frac{A}{4\pi^2}\right]^2 \int\int e^{i(\boldsymbol{q}-\boldsymbol{q}')\cdot\boldsymbol{X}} q_x q_x' h(\boldsymbol{q}) h^*(\boldsymbol{q}') d\boldsymbol{q}\, d\boldsymbol{q}'. \tag{9b}$$

Similarly, for the second-derivative term,

$$h_{xx}(\boldsymbol{X}) = \frac{A}{4\pi^2}\int e^{i\boldsymbol{q}\boldsymbol{X}}\left(-q_x^2\right)h(\boldsymbol{q})d\boldsymbol{q}. \tag{9c}$$

By substituting in the delta function arising from $\delta(\boldsymbol{q}-\boldsymbol{q}') = \int \frac{e^{i(\boldsymbol{q}-\boldsymbol{q}')X}}{4\pi^2} d\boldsymbol{X}$ and then carrying out the integral over d$\boldsymbol{X}$, we can simplify the first-order derivative terms (tension h terms) in Eq. 8 to

$$\int \left(h_x(\boldsymbol{X})^2 + h_y(\boldsymbol{X})^2\right) \mathrm{d}\boldsymbol{X} = \frac{A^2}{4\pi^2}\int \delta(\boldsymbol{q} - \boldsymbol{q}')q^2 h(\boldsymbol{q}) h^*(\boldsymbol{q}') d\boldsymbol{q}\, d\boldsymbol{q}'. \tag{10}$$

The same may be done for the second derivative terms, yielding

$$\int \left(h_{xx}(\boldsymbol{X}) + h_{yy}(\boldsymbol{X})\right)^2 d\boldsymbol{X} = \frac{A^2}{4\pi^2}\int \delta(\boldsymbol{q} - \boldsymbol{q}')q^4 h(\boldsymbol{q}) h^*(\boldsymbol{q}') d\boldsymbol{q}\, d\boldsymbol{q}'. \tag{11}$$

Taking the $d\boldsymbol{q}'$ integral cancels out the delta function terms. Placing these expressions into the Helfrich Hamiltonian from Eq. 8 and simplifying will result in

$$E = \frac{1}{2}\left[\frac{A^2}{4\pi^2}\right]\int h(\boldsymbol{q})h^*(\boldsymbol{q}')\left[\tau q^2 + \kappa_b q^4\right]d\boldsymbol{q}. \tag{12}$$

The integral of $d\boldsymbol{q}$ (the sum of oscillator modes) simplifies to $\frac{2\pi^2}{A}$, and $h(\boldsymbol{q})h^*(\boldsymbol{q}')$ can be represented as $\langle |h(q)|^2 \rangle$, which is the time-averaged q-vector (frequency) of undulations. Setting $\langle E \rangle = \frac{k_B T}{2}$ from the equipartition theorem and rearranging yields the following equation that can be solved for the bending modulus using the lowest q-vectors that contribute the most to the average q-vectors.

$$< |h(q)|^2 > = \frac{k_B T}{A(\tau q^2 + \kappa_b q^4)} \tag{13}$$

In the regime of small $\boldsymbol{q}$, the q^4 term dominates over the tension term, allowing us to neglect τq^2. A log-log plot of $\boldsymbol{q}$-vector contributions to $\langle |h(q)|^2 \rangle$ produces a linear fit that could be used to determine the bending modulus. The result of this procedure is illustrated in Fig. 3.

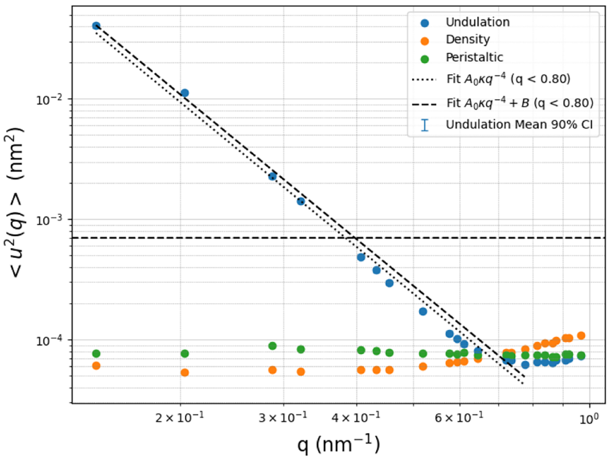

FIGURE 3 Log-log plot of q-vector contribution to $\langle |h(q)|^2 \rangle$

The second analysis method involves implementing the density correlation function within the capillary wave theory. This method, built on the work of Bedeaux and Weeks from 1985 (13) and implemented by Hernández-Muñoz et al. (12), is referred to as BW-DCF. It begins by defining the Hamiltonian of capillary wave of a fluid-fluid interface and takes a similar form to the previously seen Helfrich Hamiltonian.

$$\Delta F = \int \left\{\frac{\tau}{2}|\nabla h(\boldsymbol{r})|^2 + \frac{1}{2}mg\Delta\rho h^2(\boldsymbol{r})\right\} d\boldsymbol{r}, \tag{14}$$

where the second term represents gravitational forces but could be replaced by other external force terms on the surface of the interface. Here, $\boldsymbol{r}$ is d-dimensional and represents the projection of the surface onto the $z = 0$ (x-y) plane (15). In this context, d$\boldsymbol{r}$ can be interpreted as dx dy, making the resemblance between Eqs. 14 and 8 more visible. The height function is still present and represents the Fourier series of vibrational $\boldsymbol{q}$-vectors:

$$h(\boldsymbol{r}) = \sum_q h(\boldsymbol{q})e^{i\boldsymbol{q}\cdot\boldsymbol{r}}. \tag{15}$$

Implementing these Fourier series into the Hamiltonian of capillary wave yields

$$\Delta F = \frac{1}{2}\int \sum_{\mathrm{q}} h(\boldsymbol{q})h(\boldsymbol{q}')\left[\tau q^2 + mg\Delta\rho\right] d\boldsymbol{r}. \tag{16a}$$

Equation 16a may then be simplified further by integrating out the d$\boldsymbol{r}$ term. For a d-dimensional object with side length L, integrating out d$\boldsymbol{r}$ results in a constant multiplicative factor of L^{d-1}. Defining the capillary length $L_c = \left[\frac{\tau}{mg\Delta\rho}\right]^{\frac{1}{2}}$ transforms the Hamiltonian into

$$\Delta F = \frac{\tau}{2}L^{d-1}\sum h(\boldsymbol{q})h(\boldsymbol{q}')\left[q^2 + L_c^{-2}\right]. \tag{16b}$$

Using Gaussian fluctuation theory, the classical average is

$$\langle h(\boldsymbol{q})h(\boldsymbol{q}')\rangle = \left[\beta\tau L^{d-1}\left(q^2 + L_c^{-2}\right)\right]^{-1}, \tag{17}$$

where $\beta = (\kappa_B T)^{-1}$. From this, the height-height correlation function S is defined:

$$S(\boldsymbol{r}) = \langle h(\boldsymbol{r}+\boldsymbol{s})h(\boldsymbol{s})\rangle. \tag{18}$$

Using the above classical average, this correlation function simplifies to

$$S(\boldsymbol{r}) = \frac{1}{\beta\tau(2\pi)^{d-1}}\int \frac{e^{i\boldsymbol{r}\cdot\boldsymbol{q}}}{q^2 + L_c^{-2}}d\boldsymbol{q} \ . \tag{19}$$

Furthermore, the height distribution function for both singlets and pairs contains more information regarding height correlation:

$$P(z) = \langle \delta(h(\boldsymbol{r}) - z)\rangle \text{ and} \tag{20a}$$

$$P(z_1, z_2, r_{12}) = \langle \delta(h(\boldsymbol{r}_1) - z_1)\delta(h(\boldsymbol{r}_2) - z_2)\rangle. \tag{20b}$$

Using the work of Wang and Uhlenbeck from 1945 (21), these distribution functions may be represented by

$$P(y_1 \dots y_n) = \frac{1}{(2\pi)^n}\int \dots \int \exp\left[i\sum_1^n y_k t_k - \frac{1}{2}\sum_{k,l=1}^n b_{kl}t_k t_l\right]. \tag{21}$$

After using Gaussian integral substitutions, the distribution functions can be simplified to

$$P(z_1, z_2, r_{12}) = \frac{1}{2\pi}\left[S(0)^2 - S(r_{12})^2\right]^{\frac{1}{2}} \exp \times \left[\frac{1}{2\left[S(0)^2 - S(r_{12})^2\right]}\left[2S(r_{12})z_1 z_2 - S(0)\left(z_2^2 + z_1^2\right)\right]\right] \text{ and} \tag{22a}$$

$$P(z_1) = [2\pi S(0)]^{\frac{1}{2}} \exp\left[-\frac{z^2}{2S(0)}\right]. \tag{22b}$$

The pair distribution function can also be generalized to be

$$P(z_1, z_2, r_{12}) = \exp\left[S(r_{12})\frac{\partial^2}{\partial z_1 \partial z_2}\right] P(z_1)\, P(z_2), \tag{23}$$

where the exponential operator is to be represented by its Taylor series, leading into the second contribution for the BW-DCF analysis method by Hernández-Muñoz et al. (12). The pair distribution function, once expanded, can take the general form

$$G_{BW}(z_1, z_2, q) = \sum_{n=1}^{\infty}\frac{\widehat{S}_n(q)}{n!}\frac{d^n\rho(z_1)}{dz_1^n}\frac{d^n\rho(z_2)}{dz_2^n}, \tag{24a}$$

where $\widehat{S}_n(q) = \int d^2\boldsymbol{x}S(\boldsymbol{x})^n e^{i\boldsymbol{q}\cdot\boldsymbol{x}}$. This pair distribution function, also known as the density correlation function, now allows the expansion to desired levels of n, as well as the representation of other thermal fluctuation contribution modes. One of the modes that contributes to the fluctuations is the interlayer correlation, that is, the density correlation between the two layers of the lipid bilayer. This term is also not seen in the function where $n = 1$, but by examining the expansion further, this term plays an important role in the thermal fluctuations. This new term is known as the coupled undulation (CU). This allows the density correlation function to be rewritten for this CU term.

$$G_{BW}^{+-}(z_1, z_2, q) = \sum_{n=1}\frac{S_n^{CU}(q)}{n!}\frac{\partial^n\rho^+(z_1)}{dz_1^n}\frac{\partial^n\rho^-(z_2)}{dz_2^n}. \tag{24b}$$

To utilize the density correlation function, the densities must first be found, as well as their derivatives. To find these functions, we model the density as

$$\rho^{\pm}(z) = \frac{\rho_o \exp\left[-\frac{\left(\frac{z \pm d}{2}\right)^2}{2a}\right]}{\sqrt{2\pi a}}, \tag{25}$$

where ρ_o is the two-dimensional density, d is the thickness of the membrane, and a is the mean-square width of the density described by

$$\rho(z) = \langle\frac{1}{A_o}\sum_{i=1}^N \delta(z - z_i)\rangle. \tag{26}$$

Two matrices are defined as

$$A_{nm} = \int dz_1 \partial_n\rho^+(z_1)\partial_m\rho^+(z_1) \text{ and} \tag{27}$$

$$B_{nm}(\boldsymbol{q}) = \int dz_1 \int dz_2 \partial_n\rho^+(z_1)G^{+-}(z_1, z_2, q)\partial_m\rho^-(z_2) \tag{28a}$$

$$= \frac{1}{A_o}\langle\sum_{i=1}^{N_+}\sum_{j=1}^{N_-}\partial_n\rho^+(z_i)\cos\left(\boldsymbol{q}\cdot\left(\boldsymbol{x}_\mathbf{i} - \boldsymbol{x}_\boldsymbol{j}\right)\right)\partial_m\rho^-\left(z_j\right)\rangle. \tag{28b}$$

The first equation for B (Eq. 28a) requires the calculation of the correlation function and binning of z_1 and z_2, whereas the second equation (Eq. 28b) does not require such calculations. A final matrix is defined as

$$C_{nm} = \delta_{nm}\frac{S_n^{CU}(q)}{n!}. \tag{29}$$

This C matrix can be represented by the A and B matrices through Eq. 24b and results in the following identity:

$$\frac{k_B T}{q^2\gamma^{CU}(q)} = \sum_{n,m=1}^{n_{BW}} A_{1n}^{-1}B_{nm}(q)A_{m1}^{-1}, \tag{30}$$

where n_{BW} is the truncation order of the Taylor expansion of the correlation function and γ_{cu} is a function that represents the coupled-undulatory q-dependent surface tension and is fit to the data. An example is a quadratic

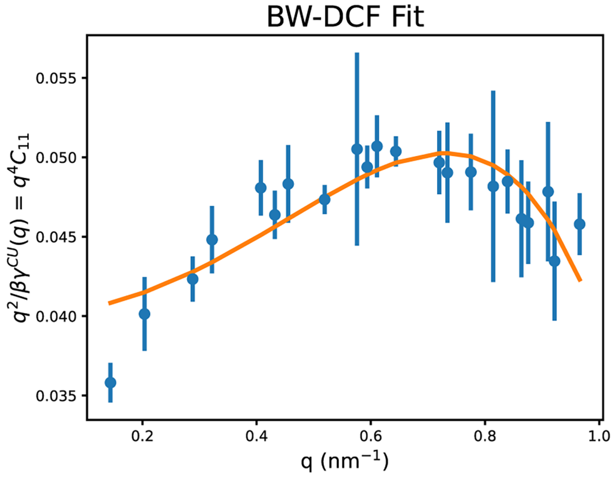

FIGURE 4 A representative plot of the quadratic fit of the coupled-undulatory q-dependent surface tension (γ_{cu}), used within the BW-DCF method

fit, which allows the following representation and, thus, for the bending modulus to be extracted. A few other examples of the representation of γ can be found in Hernández-Muñoz et al. (6). The results of this fitting can be seen in Fig. 4.

$$\gamma_{cu} = \gamma_o + \kappa_b q^2 \tag{31}$$

The final method considered is the RSF approach (6–10), which tracks local fluctuations across the membrane surface, yielding spatially resolved values of both bending and tilt moduli. RSF provides mechanistic insights into how molecular architecture and membrane geometry modulate membrane mechanics, which cannot be directly revealed by the global fluctuation spectral fits. This method utilizes the tilt of lipids in reference to the local water-membrane interface to compute the membrane bending modulus. It is first assumed that the elastic energy of curvature is a quadratic function of the local area normal derivatives (3). Keeping the z axis fixed, the free energy of deformation can be defined as

$$f_s = \frac{1}{2} K_c \langle \nabla \boldsymbol{n} - \nabla \boldsymbol{N} \rangle^2, \tag{32}$$

where $\boldsymbol{n}$ represents the lipid tilt director, $\boldsymbol{N}$ is the local normal to the interface, and f_s is an energy per unit area, representing the free energy of deformation. Both $\boldsymbol{n}$ and $\boldsymbol{N}$ are two-dimensional ($d = 2$). The term $\langle \nabla \boldsymbol{n} - \nabla \boldsymbol{N} \rangle$ is known as the splay, denoted as S_t. The splay of a patch in the membrane is considered an independent degree of freedom, exhibiting a Boltzmann distribution (8), which allows

$$P(S_t) = C \exp\left(\frac{-K_c A_l (\nabla \boldsymbol{n} - \nabla \boldsymbol{N})^2}{2 k_B T} \right), \tag{33a}$$

where A_l is the area of a lipid and arises due to f_s representing the energy per unit area. Fig. 5 shows a visual representation of lipid splay.

This equation can be solved to find the splay modulus, that is,

$$-\frac{2 k_B T}{A_l} \ln P(S_t) = K_c (\nabla \boldsymbol{n} - \nabla \boldsymbol{N})^2. \tag{33b}$$

This equation allows the splay modulus (or bending modulus) to be extracted once the splay is known. To determine the splay, consider the divergence of a vector field $\boldsymbol{v}$ at a point $\boldsymbol{p}$ on the surface (8):

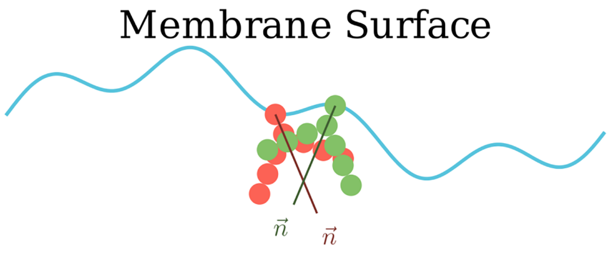

FIGURE 5 Representation of splay between two lipids

$$\boldsymbol{v}(\boldsymbol{p}) = \sum_{i=1}^{3} \nu_i(\boldsymbol{p}) \boldsymbol{e}_i. \tag{34}$$

The vectors $\boldsymbol{e}_1$ and $\boldsymbol{e}_2$ are orthogonal unit vectors tangent to the surface and $\boldsymbol{e}_3$ is the normal to the surface. Utilizing finite differences,

$$\nabla \boldsymbol{v}(\boldsymbol{p}) = \frac{\sum_{i=1}^{2} \left(\nu_i(\boldsymbol{p} + h\boldsymbol{e}_i) - \nu_i(\boldsymbol{p}) + \nu_i(\boldsymbol{p}) - \nu_i(\boldsymbol{p} - h\boldsymbol{e}_i) \right)}{2h}. \tag{35a}$$

Once expanded, this equation simplifies to

$$\nabla \mathbf{v}(\boldsymbol{p}) = \frac{1}{2} \left[\nu_1' + \nu_{-1}' + \nu_2' + \nu_{-2}' \right]. \tag{35b}$$

Hence, the splay is

$$S_t = \frac{1}{2} [S_1 + S_{-1} + S_2 + S_{-2}], \tag{36a}$$

where

$$S_i = \frac{n_i(\boldsymbol{p} + h\mathbf{e}_i) + N_i(\mathbf{p} + h\boldsymbol{e}_i) - n_i(\mathbf{p}) - N_i(\mathbf{p})}{h}. \tag{36b}$$

This allows the splay for lipids to be determined, which in turn can be used to construct the probability distribution. Finally, the quadratic fit to Eq. 33b can be used to determine the bending modulus of the monolayer being analyzed. Doubling that value yields the bilayer bending modulus (7). This quadratic fit can be seen in Fig. S1.

Whereas our RSF implementation follows the original Doktorova and Khelashvili (10) protocol, alternative approaches exist, such as the real space instantaneous surface method by Allolio et al. (22), accompanied by a publicly available Lipidator Toolkit (https://github.com/allolio/lipidator-toolkit). The authors emphasize several advantages to the real space instantaneous surface, including spatial locality and robustness with respect to the size of the simulated membrane patch.

In multicomponent systems, the splay moduli are computed for each pair of lipids present, which are in turn used to compute the overall modulus:

$$\frac{1}{K_c} = \frac{1}{\phi_{total}} \sum_{i,j} \frac{\phi_{ij}}{\chi_{12}^{ij}}, \tag{37}$$

where ϕ_{ij} is the number of lipid pairs of species ij and χ represents the corresponding bending modulus. ϕ_{total} is the total number of pairs (8).

Equations 38 and 39 display how lipid and membrane geometrical properties are determined from the simulation trajectory. The membrane bilayer thickness, D_B, is calculated through the averaging of top and bottom z

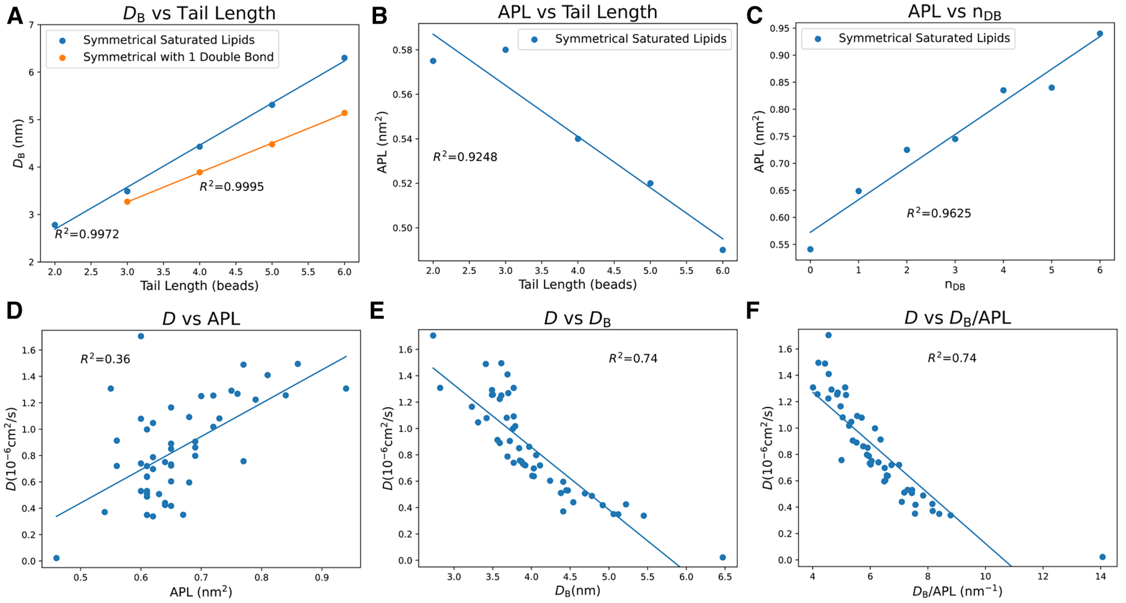

FIGURE 6 (*A*) Tail length (in beads) versus average membrane thickness for symmetrical tailed membranes. (*B*) Tail length versus APL for saturated lipids. (*C*) APL versus number of double bonds (n_{DB}). (*D*) Plot of APL versus diffusion. (*E*) Graph of diffusion versus thickness of 51 membranes. (*F*) Diffusion versus thickness/APL ratio.

coordinates across all frames, as shown in Eq. 38. The APL values are determined by dividing the square of the cell length (*x*-*y* cross-sectional area) by the number of lipids present in a monolayer, as shown in Eq. 39.

$$D_B = \langle \langle z_{top} \rangle_{frame} - \langle z_{bottom} \rangle_{frame} \rangle_{all\ frames} \tag{38}$$

$$APL = \frac{1}{2}\left(\frac{l_{cell}^2}{n_{lipids}}\right) \tag{39}$$

RESULTS AND DISCUSSION

We conducted MD simulations of a variety of symmetrical lipid bilayers, as detailed in the computational details section. The simulations were designed to evaluate key membrane properties, focusing on the effects of tail length and the presence of double bonds in the lipid tail.

We first examined the effect of the number of beads and double bonds on lipid geometrical parameters such as APL and membrane thickness (D_B).

Fig. 6 *A* shows the impact of the lipid tail length (number of beads) on membrane thickness for symmetrically tailed membranes (labeled with a D as the first letter), including both saturated lipids and those with a double bond in each tail. As beads are added to the tail, the membrane thickness increases linearly. Introducing a double bond into each tail (i.e., replacing a bead with one representing a double bond) decreases the thickness compared to the corresponding saturated lipids. This trend remains linear but with a lower slope.

Fig. 6 *B* illustrates the linear decrease in APL, ranging from 0.58 to 0.49 nm^2, as the number of beads in saturated lipid tails increases from two to six. This reduction likely arises from decreased tail flexibility in longer lipids, which limits the ability of lipids to adopt larger cross-sectional configurations.

We compared these APL and membrane thickness values with the experimentally determined full lipid bilayer thickness, or Luzzati thickness, values from Kučerka (23) using small-angle neutron scattering and x-ray scattering, as shown in Table 2. The experimental data set includes seven lipids (DLPC, DMPC, DPPC, DSPC, POPC, SOPC, and DphyPC), measured at 30°C, which correspond to our simulations of three lipids, SOPC, DMPC, and DSPC, at 37°C using the MARTINI CG force field. Another work of Kučerka (24) also allows us to compare DGPC (di-$C_{20:1}$ or

TABLE 2 Comparison of computed thickness, D_B, and area per lipid to the experimental values determined with the small-angle neutron scattering and x-ray scattering experiments

	D_B (Å)		APL (Å^2)	
Lipid	Expt.	CG	Expt.	CG
POPC/SOPC[a]	39.1/40.0	38.9	64.3/65.5	65.0
DLPC/DMPC[a]	32.6/36.7	34.2	60.8/59.9	60.0
DPPC/DSPC[a]	Gel/Gel[c]	41.1	Gel/Gel[c]	61.0
DGPC[b]	42.5/46.4	44.1	66.6/65.7	68.0
DNPC[b]	52.2	50.6	62.7	67.0

[a]Kučerka (23).
[b]Kučerka (24).
[c]The results for DPPC/DSPC are not present since they were in a gel phase in experiment.

TABLE 3 Comparison of the results of the CG-MD simulations of POPC and POPE membranes in this study to the AA-MD results

Lipid	All-atom D_B (Å)	MARTINI D_B (Å)	All-Atom APL (Å^2)	MARTINI APL (Å^2)
POPC[a]	37.5	38.9	63.1	65.0
POPE[a]	43.4	40.3	55.6	61.0

[a]Shahane (25).

di-$C_{22:1}$) and DNPC (di-$C_{24:1}$) membranes, also at 30°C (Table 2). The results show a good agreement between the experimental data and our CG dynamics simulations for POPC/SOPC and DLPC/DMPC lipids. Thickness for DGPC aligns well with experiments, though APL showed a slight deviation. For DNPC, the computed thickness is in reasonable agreement with the experiment, whereas the APL differs by 4.3 Å^2.

The APL and thickness data for POPC and POPE can be compared to AA results by Shahane (25) (see Table 3). The AA simulations were performed at 303.15 K for POPC and at 310 K for POPE, matching the MARTINI simulation temperature in the latter case. The results show good agreement between AA and CG results for POPC membranes, whereas for POPE membranes, the CG model predicts a slightly decreased thickness and increased APL, as compared with the AA simulations.

Next, Fig. 6 *C* shows that increasing the number of double bonds increases the APL. This is consistent with previous findings (Demel (26), Evans (27), and Hyvönen (28)) and can be attributed to the "kink" caused by double bonds, which occupies more lateral space within the membrane.

A key defining trait of membranes is the lateral diffusion of lipids within the membrane. Fig. 6 *D* shows the plot of APL versus the two-dimensional diffusion coefficient, featuring a weak increasing trend, with a large fraction of membranes having an APL between 0.6 and 0.7 nm^2. Comparison of the membrane thickness with the corresponding diffusion coefficient of constituent lipids exhibits a prominent inverse trend (Fig. 6 *E*). The thickness of the membrane was also compared to the APL over the diffusion coefficient ratio. The corresponding Fig. 6 *F* shows an apparently linear trend with an R^2 of 0.74 (0.85 after the DXPE data point removed; see the discussion on DXPE below).

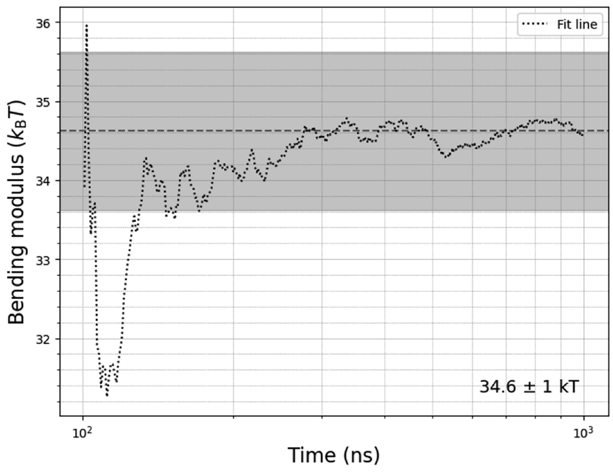

FIGURE 7 Bending modulus as function of the simulation time used for q^{-4} analysis of POPC membrane (40 × 40 × 20 nm box)

Encouraged by the shown above good consistency of the membrane dynamics from our MD simulations with literature data, we proceeded with establishing the proper simulation and analysis parameters for determining the bending modulus of membranes. First, we validated that the selected time window of 1 μs for the MD simulations is sufficient for convergence of the bending modulus. To do this, the bending modulus was computed for the POPC MD trajectory as a function of time, where time represents the end time point of the analysis, whereas the start point was always the beginning of the simulation. The corresponding Fig. 7 shows that the bending modulus converges to a value within k_BT within approximately 50–100 ns; however, longer timescales (typically up to 400 ns) may be necessary for the convergence of the bending modulus of other systems. A similar analysis was also performed for all model systems to validate the convergence.

To examine the replicability of the experiments, 5 simulations of a 40 × 40 nm POPC membrane were performed using a total box thickness of 20 nm. All runs were generated the same way, with the only difference between them being the randomly generated initial velocity vectors. The resulting bending modulus values were very similar, with the standard variation within 1%–1.5% (or 0.4 k_BT) (Table S1), showing that the MD simulations are replicable.

Our in-house implementation of the three analysis methods, introduced above, was validated through comparison to the published work from Brandt (11), Hernández-Muñoz (12) and Khelashvili (10). An 8192 total lipid system of DMPC (encoded as DLPC in MARTINI) was run at 300 K, and a bending modulus of 35.17 k_BT was found through the q^{-4} method, agreeing with the result of 36.21 k_BT (15 × 10^{-20} J) produced by Brandt (11). The BW-DCF method was examined through the simulation of POPC, where Hernández-Muñoz (12) ran a 6000 total lipid simulation and found the result of 25.7 ± 2.0 k_BT, agreeing well with our result of 23.69 kT found in this paper. Finally, for RSF, a simulation of 416 total atomistic POPC at 25°C was run to compare to the published work of Doktorova and Khelashvili (10). The results from their 2017 paper (10) revealed a bending modulus of 24.3 k_BT, whereas our results found a bending modulus of 12.12 k_BT for the monolayer, which turns out to be 24.24 k_BT for the bilayer. These results conclude that the methods of analysis are accurate to the published findings.

Next, we investigated the variable parameters of the membrane systems, these being salt, temperature, pressure, and box size. Simulations using salt concentrations of 0.05,

TABLE 4 Bending modulus values in k_BT of POPC membranes at varying box sizes

Box size (x, y, z; nm)	κ (q^{-4}) (k_BT)	κ (BW-DCF) (k_BT)	κ (RSF) (k_BT)
15 × 15 × 20	41.00	25.2	25.51
20 × 20 × 20	37.09	23.7	25.09
30 × 30 × 20	34.01	23.38	25.05
40 × 40 × 10	33.06	23.4	24.53
40 × 40 × 12.5	34.12	24.41	24.6
40 × 40 × 15	34.16	24.12	24.7
40 × 40 × 17.5	33.81	24.73	24.64
40 × 40 × 20	34.86	24.75	24.63
50 × 50 × 20	33.25	25.05	24.16
60 × 60 × 20	33.25	26.72	22.99

0.1, and 0.15 mM NaCl show that the bending modulus does not change much, with values of 30.19, 30.38, and 29.9 k_BT, respectively. Pressure was varied by 0.05 bar in both directions, and the bending modulus did not change. Table 4 shows the results of varying the box size on the bending modulus of POPC membranes. The results show that increasing the box thickness (z) does not result in a large change in the bending modulus in any of the analysis methods. Reducing the box lateral dimensions (x-y) results in an increase of the bending modulus in the q^{-4} method, the effects of which seem to be negligible for box sizes with a side larger than 30 nm. The other bending modulus values remain largely unaffected by the x-y dimension change. The data of Table 4 are visualized in Fig. 8, which displays the optimal x-y box dimensions at 30+ nm to reduce artifacts due to the finite-size effect.

The effect of the thermostat was investigated by simulating POPC membranes at 310 K using Berendsen (29), Nose-Hoover (30,31), and V-rescale (32) thermostat models implemented in GROMACS (15) The results of the simulations concluded that these three thermostats produce nearly identical results, i.e., 34.33, 34.01, and 33.94 k_BT for Berendsen, Nose-Hoover, and V-rescale thermostats, respectively. These bending modulus values being within 5% of each other.

To investigate the effect of temperature on the system, POPC membranes were also run at various temperatures in the range from 290 to 320 K. The results of these trials can be seen in Table 5. Examining the results expectedly shows the decreased bending modulus with increased temperature by about 1–2 k_BT per 10°.

Table 6 shows the statistics of the bending modulus for each lipid headgroup. The data indicate that sphingomyelin (SM) membranes are more rigid compared to phosphatidylcholine (PC) and phosphatidylethanolamine (PE) membranes. Among the latter two, PE membranes are less rigid according to the q^{-4} and BW-DCF approaches. The RSF approach presents conflicting results, suggesting that PE membranes are the most rigid, whereas the PC membranes are the least rigid. This discrepancy likely arises from a few simulations with elevated RSF values, such as DTPE, DLPE, and DYPE, which deviate from the trends observed in Table S2. Furthermore, Table 6 reveals differences in the variation of bending moduli among the headgroups, with SM membranes displaying a narrower spread compared to the PC and PE membranes. The high variability in PE membranes can be attributed to outliers, and removing those anomalies aligns their deviation with that of PC membranes.

Fig. 9 illustrates the bending moduli of PC, PE, and SM membranes composed of saturated, symmetric lipids with varying bead lengths. The BW-DCF method shows an increasing trend in the bending modulus as the number of beads increases in all three head types. For PC and SM membranes, the RSF and q^{-4} methods do not exhibit such trends, with the RSF staying flat, whereas the q^{-4} method depicts a parabolic trend. In the case of PE, we see a large jump at $n = 6$ beads, corresponding to DXPE. DXPE exhibits signs of being in a different phase, having a low APL value, high thickness, and low diffusion coefficient. The trends of a lower APL, higher thickness, and lower diffusion coefficient are associated with a membrane being in the gel phase (33). To further examine this point, the radial distribution function of DXPE was compared to POPC and can be found in Fig. S5. DXPE exhibited sharpened peaks compared to POPC's more broad peaks, meaning DXPE

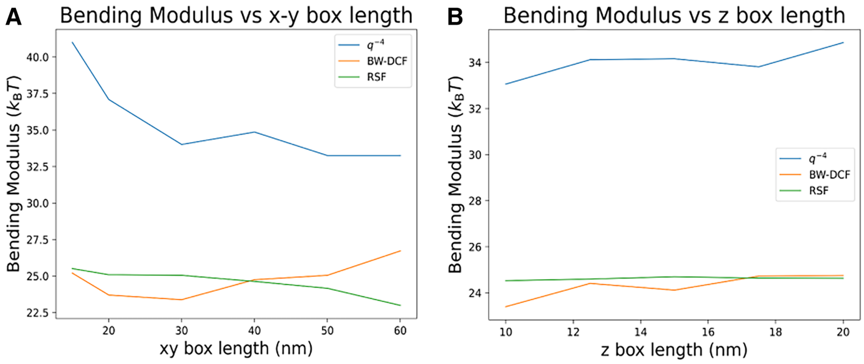

FIGURE 8 Effect of the lateral (A) and normal (B) box dimensions of bending modulus of POPC membranes

TABLE 5 Bending modulus in $k_B T$, with the values of J shown in parentheses, of POPC membranes at various temperatures

T, K	$\kappa(q^{-4})$	κ (BW-DCF)	κ (RSF)
290	38.42 (1.54×10^{-19})	29.01 (1.16×10^{-19})	27.33 (1.09×10^{-19})
300	36.62 (1.52×10^{-19})	27.06 (1.12×10^{-19})	25.61 (1.06×10^{-19})
310	34.27 (1.47×10^{-19})	25.84 (1.11×10^{-19})	24.57 (1.05×10^{-19})
320	32.09 (1.42×10^{-19})	23.69 (1.05×10^{-19})	23.41 (1.03×10^{-19})

is in a more ordered phase, associated with gel-phased membranes.

Fig. 10, *A–C*, compares the bending moduli of the three previously introduced methods for single-component membranes in a pairwise fashion. The RSF method demonstrates a weak linear correlation with both BW-DCF and q^{-4} methods ($R^2 = 0.51$ in both cases). The plot of q^{-4} with BW-DCF shows a stronger linear correlation ($R^2 = 0.82$).

Another important aspect explored is the relationship between geometrical lipid properties and the bending modulus. Fig. 10, *D–F*, shows the bending modulus against the APL for single-component membranes. The results suggest a possible trend of a decreasing bending modulus with an increasing APL, which could be explained by the idea that a higher APL may reduce the density of lipids in a given area, potentially weakening long-range interactions and lowering the energy required for deformation. Exceptions occur in the range of 0.6–0.7 nm^2, where q^{-4} and BW-DCF methods show insensitivity to the APL.

Fig. 10, *G–I*, displays the bending modulus as a function of membrane thickness. Inspecting these graphs shows that a large portion of analyzed membranes have a thickness between 3.5 and 4 nm. The bending modulus increases with thickness. This trend is further supported by Fig. S2, which plots thickness against APL with color-coded bending modulus values, revealing additional potential relationships.

The ratio of membrane thickness to APL provides another lens through which to understand the bending modulus. Fig. 11, *A–C*, shows a linear relationship between the bending modulus and the thickness/APL ratio in q^{-4} and BW-DCF methods. From a theoretical mechanics perspective, the relationship is logical: a higher thickness implies that more beads are present in the lipid structure, necessitating greater energy for deformation. A lower APL indicates increased intermolecular interactions taking place, also requiring more energy for distortion.

Lastly, Fig. 11, *D–F*, investigates the relationship between lateral diffusion and bending modulus values. For the q^{-4} and BW-DCF methods, the data reveal an interesting relationship: as diffusion increases, the bending modulus decreases. This observation aligns with the expectation that greater lateral lipid movement corresponds to reduced rigidity in the membrane structure.

TABLE 6 Aggregated mean and standard deviation values in $k_B T$ of the bending moduli among PC, PE, and SM headgroups

Headgroup	$\kappa(q^{-4})$	κ(BW − DCF)	κ(RSF)
PC	29.0 ± 9.0	23.5 ± 11.4	23.0 ± 5.7
PE	28.1 ± 14.0	19.2 ± 17.2	32.6 ± 26.6
PE[a]	26.0 ± 10.6	15.9 ± 9.3	27.1 ± 10.4
SM	35.7 ± 4.0	34.9 ± 8.6	26.4 ± 2.6

[a]DXPE removed; see discussion in the main text.

CONCLUSION

Membranes exhibit fascinating mechanical properties, making them a subject of interest for many years across various scientific disciplines. Despite their significance, the bending properties of membranes remain relatively unexplored, both experimentally and computationally. Discrepancies in the reported bending modulus values often arise due to differences in the computational analysis method or experimental technique. Advancing our understanding of the membrane bending modulus is critical for studying membrane-associated systems, including transmembrane protein complexes, and for elucidating the fundamental biophysical properties of cellular membranes.

CG-MD simulations provide a powerful platform for investigating the bending properties of membranes. The computational efficiency of CG-MD, combined with readily available tools for generating input structures, makes it a rapid and effective approach for exploring membrane mechanics. These simulations allow for the systematic evaluation of key membrane properties that are otherwise challenging to measure experimentally.

Several methodologies are employed to analyze MD simulation data to estimate the bending modulus, a parameter directly linked to the membrane's energy of deformation. When considering all lipids included in our study, the BW-DCF method (12,13) consistently yields lower bending modulus values compared to the RSF (6–10) and q^{-4} (11) methods. A strong linear correlation is observed between the q^{-4} and BW-DCF methods, whereas the RSF method produces higher modulus values and lacks linear correlation with the other two approaches.

CG-MD simulations also reveal meaningful relationships between membrane geometry and mechanical properties. Notably, the ratio of membrane thickness to APL was shown to be related to the bending modulus of membranes determined by BW-DCF and q^{-4} methods. Additionally, this ratio exhibited an inverse relationship with lateral diffusion, highlighting the intricate interplay between membrane structure and dynamics.

These findings provide a foundation for leveraging modern data-driven approaches, such as machine learning, to further explore membrane mechanics. By incorporating these insights, future studies can develop computationally efficient methods to predict membrane properties, even for systems that are challenging to stabilize in traditional

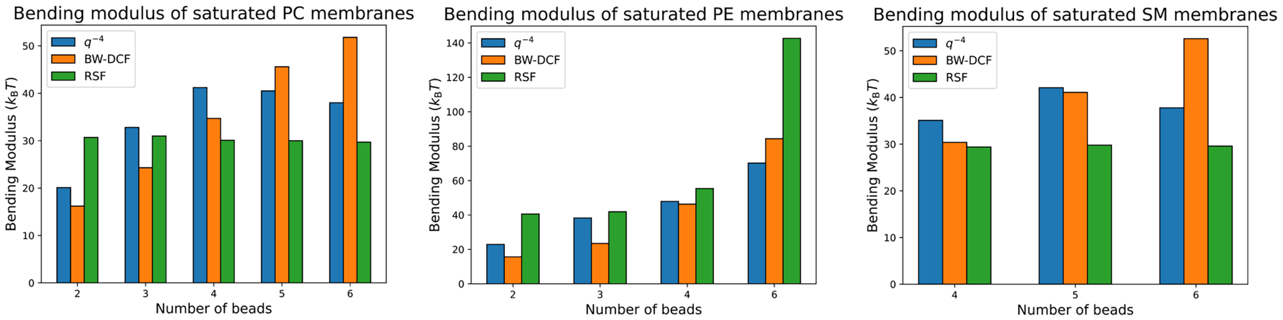

FIGURE 9 The bending modulus, predicted by three approaches, as a function of the length of saturated symmetric lipids, expressed as number of beads in the MARTINI CG model

simulations. This integration of computational modeling with advanced analytical techniques holds great promise for deepening our understanding of membrane behavior and expanding the scope of membrane biophysics research.

ACKNOWLEDGMENTS

This work is supported by the National Science Foundation under grant CMMI-2011220. The MD simulations were partially conducted using the resources provided by ACCESS CI, BIO230197. The authors would like

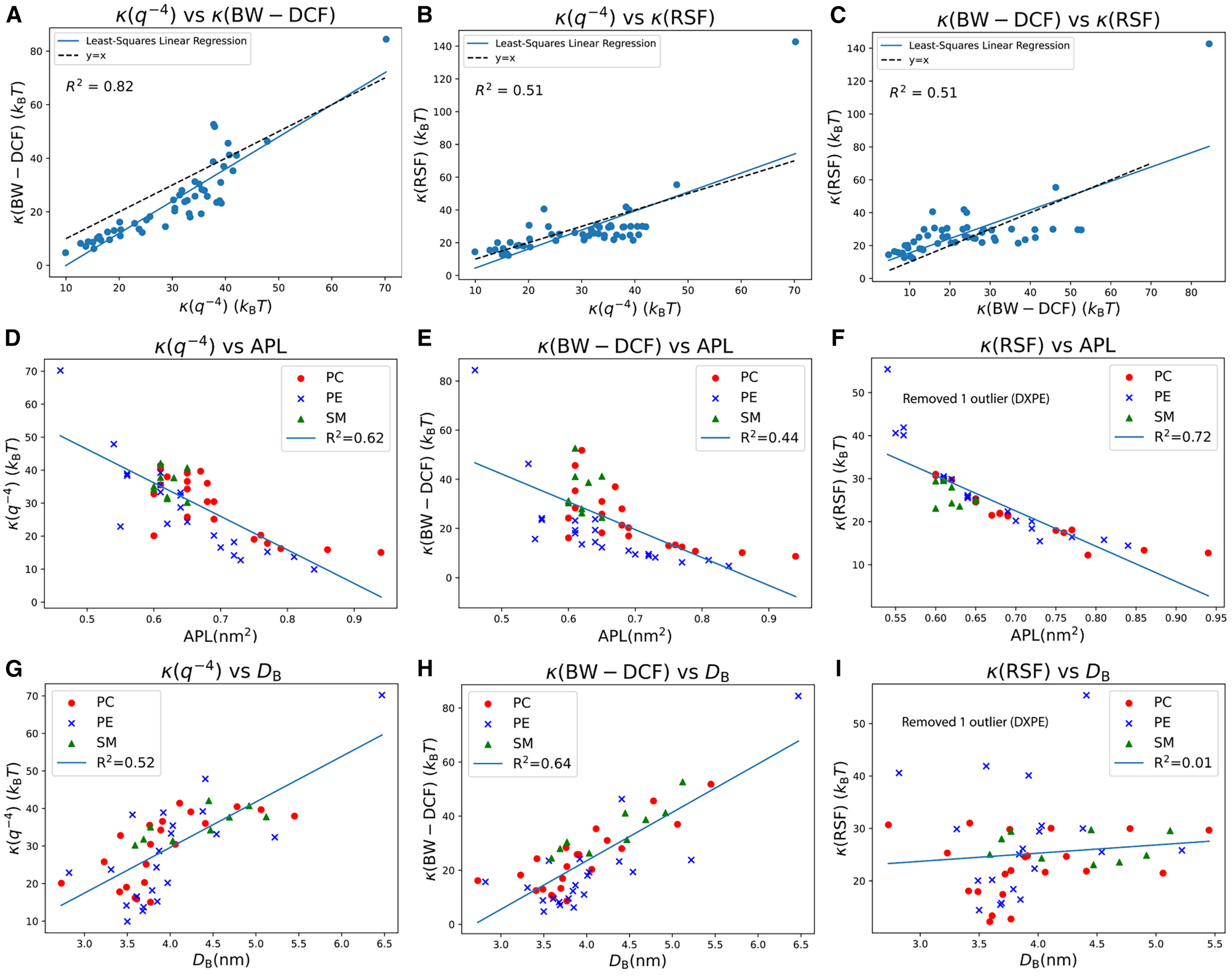

FIGURE 10 Top row: pairwise linear regressions for the bending moduli obtained (*A*) q^{-4} versus BW-DCF, (*B*) q^{-4} versus RSF, and (*C*) BW-DCF versus RSF for 51 membranes. Middle row: plots of bending modulus versus area per lipid (APL) obtained using (*D*) q^{-4}, (*E*) BW-DCF, and (*F*) RSF. Bottom row: plot of bending modulus versus average membrane thickness obtained using (*G*) q^{-4}, (*H*) BW-DCF, and (*I*) RSF.

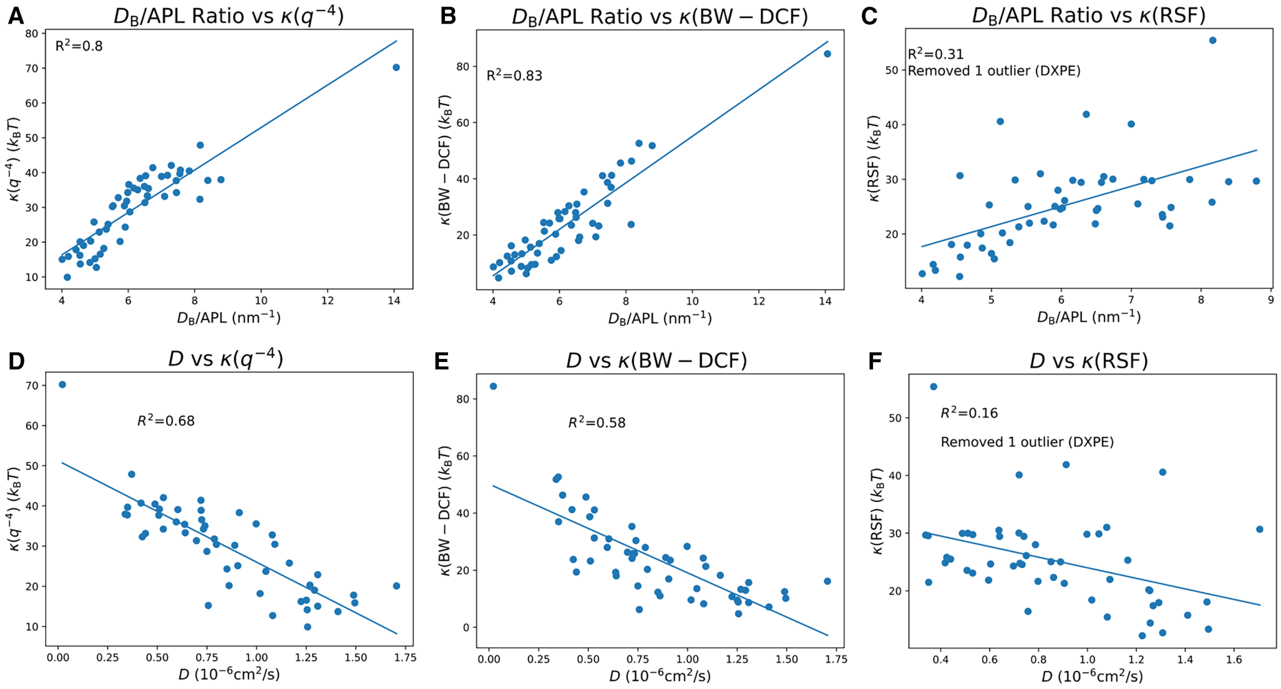

FIGURE 11 The (*A–C*) D_B/APL ratio versus bending modulus and (*D–F*) diffusion coefficient versus bending modulus values for the membranes simulated in this study.

to thank Arslan Atangulov for helpful discussions and assisting with the preparation of Fig. 1. During the preparation of this work, the authors used ChatGPT-4o to obtain suggestions for improving the manuscript readability. After using this tool/service, the authors reviewed and edited the content as needed and take full responsibility for the content of the publication.

AUTHOR CONTRIBUTIONS

M.R.T. conceptualized and designed the study, performed data analysis, and drafted the manuscript. J.P. carried out all-atom molecular dynamics simulations, conducted preliminary analyses and coarse-grained simulations, and contributed to an early draft of the manuscript. S.B. performed coarse-grained simulations, analyzed the results, and contributed to manuscript writing.

DECLARATION OF INTERESTS

The authors declare no competing interests.

SUPPORTING MATERIAL

Supporting material can be found online at https://doi.org/10.1016/j.bpj.2025.07.014.

REFERENCES

1. Zhukov, A., and V. Popov. 2023. Eukaryotic Cell Membranes: Structure, Composition, Research Methods and Computational Modelling. *Int. J. Mol. Sci.* 24:11226.
2. Pinigin, K. V. 2022. Determination of Elastic Parameters of Lipid Membranes with Molecular Dynamics: A Review of Approaches and Theoretical Aspects. *Membranes.* 12:1149.
3. Helfrich, W. 1973. Elastic Properties of Lipid Bilayers: Theory and Possible Experiments. *Z. Naturforsch. C Biosci.* 28:693–703.
4. Jiang, W., J. S. Del Rosario, …, Y. L. Luo. 2021. Crowding-induced opening of the mechanosensitive Piezo1 channel in silico. *Commun. Biol.* 4:84.
5. Nagle, J. F., M. S. Jablin, …, K. Akabori. 2015. What are the true values of the bending modulus of simple lipid bilayers? *Chem. Phys. Lipids.* 185:3–10.
6. Khelashvili, G., G. Pabst, and D. Harries. 2010. Cholesterol orientation and tilt modulus in DMPC bilayers. *J. Phys. Chem. B.* 114:7524–7534.
7. Khelashvili, G., B. Kollmitzer, …, D. Harries. 2013. Calculating the bending modulus for multicomponent lipid membranes in different thermodynamic phases. *J. Chem. Theor. Comput.* 9:3866–3871.
8. Johner, N., D. Harries, and G. Khelashvili. 2014. Curvature and lipid packing modulate the elastic properties of lipid assemblies: Comparing HII and lamellar phases. *J. Phys. Chem. Lett.* 5:4201–4206.
9. Khelashvili, G., and D. Harries. 2013. How cholesterol tilt modulates the mechanical properties of saturated and unsaturated lipid membranes. *J. Phys. Chem. B.* 117:2411–2421.
10. Doktorova, M., D. Harries, and G. Khelashvili. 2017. Determination of bending rigidity and tilt modulus of lipid membranes from real-space fluctuation analysis of molecular dynamics simulations. *Phys. Chem. Chem. Phys.* 19:16806–16818.
11. Brandt, E. G., A. R. Braun, …, O. Edholm. 2011. Interpretation of fluctuation spectra in lipid bilayer simulations. *Biophys. J.* 100:2104–2111.
12. Hernández-Muñoz, J., F. Bresme, …, E. Chacón. 2022. Bending Modulus of Lipid Membranes from Density Correlation Functions. *J. Chem. Theor. Comput.* 18:3151–3163.
13. Bedeaux, D., and J. D. Weeks. 1985. Correlation functions in the capillary wave model of the liquid–vapor interface. *J. Chem. Phys.* 82:972–979.
14. De Jong, D. H., G. Singh, …, S. J. Marrink. 2013. Improved parameters for the martini coarse-grained protein force field. *J. Chem. Theor. Comput.* 9:687–697.
15. Abraham, M. J., T. Murtola, …, E. Lindahl. 2015. GROMACS: high performance molecular simulations through multi-level parallelism

from laptops to supercomputers. *SoftwareX.* 1-2:19–25. http://dx.doi.org/10.1016/j.softx.2015.06.001.

16. Wassenaar, T. A., H. I. Ingólfsson, …, S. J. Marrink. 2015. Computational lipidomics with insane: A versatile tool for generating custom membranes for molecular simulations. *J. Chem. Theor. Comput.* 11:2144–2155.
17. browns72, Brown, S. 2025. Zenodo Repository Browns72/membrane_methods. release-zenodo (release-zenodo).
18. Gowers, R., M. Linke, …, O. Beckstein. 2016. MDAnalysis: A Python Package for the Rapid Analysis of Molecular Dynamics Simulations. *In* PROC. OF the 15th PYTHON IN SCIENCE CONF, pp. 98–105.
19. Michaud-Agrawal, N., E. J. Denning, …, O. Beckstein. 2011. MDAnalysis: A toolkit for the analysis of molecular dynamics simulations. *J. Comput. Chem.* 32:2319–2327.
20. Boal, D. 2012. Mechanics of the Cell. Cambridge University Press.
21. Wang, M. C., and G. E. Uhlenbeck. 1945. On the Theory of the Brownian Motion II. *Rev. Mod. Phys.* 17:323–342.
22. Allolio, C., A. Haluts, and D. Harries. 2018. A local instantaneous surface method for extracting membrane elastic moduli from simulation: Comparison with other strategies. *Chem. Phys.* 514:31–43.
23. Kučerka, N., M. P. Nieh, and J. Katsaras. 2011. Fluid phase lipid areas and bilayer thicknesses of commonly used phosphatidylcholines as a function of temperature. *Biochim. Biophys. Acta Biomembr.* 1808:2761–2771.
24. Kučerka, N., J. Gallová, …, J. Katsaras. 2009. Areas of monounsaturated diacylphosphatidylcholines. *Biophys. J.* 97:1926–1932.
25. Shahane, G., W. Ding, …, M. Orsi. 2019. Physical properties of model biological lipid bilayers: insights from all-atom molecular dynamics simulations. *J. Mol. Model.* 25:76.
26. Demel, R. A., W. S. M. Geurts van Kessel, and L. L. M. van Deenen. 1972. The properties of polyunsaturated lecithins in monolayers and liposomes and the interactions of these lecithins with cholesterol. *Biochim. Biophys. Acta Biomembr.* 266:26–40.
27. Evans, R. W., M. A. Williams, and J. Tinoco. 1987. Surface areas of 1-palmitoyl phosphatidylcholines and their interactions with cholesterol. *Biochem. J.* 245:455–462.
28. Hyvönen, M. T., and P. T. Kovanen. 2005. Molecular dynamics simulations of unsaturated lipid bilayers: Effects of varying the numbers of double bonds. *Eur. Biophys. J.* 34:294–305.
29. Berendsen, H. J. C., J. P. M. Postma, …, J. R. Haak. 1984. Molecular dynamics with coupling to an external bath. *J. Chem. Phys.* 81:3684–3690.
30. Nosé, S. 1984. A molecular dynamics method for simulations in the canonical ensemble. *Mol. Phys.* 52:255–268.
31. Hoover, W. G. 1985. Canonical dynamics: Equilibrium phase-space distributions. *Phys. Rev.* 31:1695–1697.
32. Bussi, G., D. Donadio, and M. Parrinello. 2007. Canonical sampling through velocity rescaling. *J. Chem. Phys.* 126:014101.
33. Kumar, A., and S. Daschakraborty. 2023. Anomalous lateral diffusion of lipids during the fluid/gel phase transition of a lipid membrane. *Phys. Chem. Chem. Phys.* 25:31431–31443.

Biophysical Journal, Volume *125*

Supplemental information

Comparative analysis of bending moduli in one-component membranes via coarse-grained molecular dynamics simulations

Sam Brown, Jessica Pallarez, and Marat R. Talipov

Supporting Information

Comparative Analysis of the Bending Modulus for Phosphatidylcholine, Phosphatidylethanolamine, and Sphingomyelin Membranes Based on the Coarse-Grained Molecular Dynamics Simulations

Sam Brown, Jessica Pallarez, Marat R. Talipov*

Table of Contents

SI 1. Membrane Generation and Run Protocol 1

SI 2. GROMACS MDP File Parameters 1

SI 3. GROMACS Mean Square Displacement and Diffusion Constant Determination 4

Table S1. Replicability of experiments; Statistics of 5 trials of 100% POPC 4

Figure S1. Quadratic fit of splay to probability density for RSF bending modulus 4

Figure S2. Color coded bending modulus of Thickness vs APL graph 5

Figure S4. Coarse-grained lipids, tail groups and head groups 6

Table S2. Lipid geometrical properties, bending modulus and diffusion from Coarse-Grained Molecular Dynamics Simulations 10

Figure S5. Radial distribution function of POPC and DXPE 12

SI 1. Membrane Generation and Run Protocol.

To generate the membranes for simulations, a python program INSANE was utilized [(https://github.com/Tsjerk/Insane; Wassenaar, et al. 2015]. This program is quite versatile for generation of membranes and membrane systems, allowing many parameters to be defined such as salts, solvent, and embedding proteins into membrane systems. A representative command line for invoking INSANE had the following form: ‘python2.7 insane.py -l DNPE:1 -salt 0.15 -x 40 -y 40 -z 20 -pbc cubic -sol W -o system.gro -p system.top -a 0.55’.

Once the membranes were generated, they are then indexed utilizing GROMACS make_ndx interactive command, fully automated with the ‘expect’ command (https://www.man7.org/linux/man-pages/man1/expect.1.html). After the indexes are generated, the membranes are run through a 2-step preparation process that includes a 10,000-step conjugate gradient minimization and 1,000,000 step NTP equilibration process at 310 K and 1 bar. After equilibration, the systems were subjected to the production step. The parameters used for the minimization, equilibration and production steps can be found in SI 2. The commands used to execute all the molecular dynamics processes (minimization, equilibration and production) are similar, and follow the format: ‘gmx grompp -f 0-min.mdp -c system.gro -r system.gro -p system.top -o 00-min.tpr’ and ‘gmx mdrun -deffnm 00-min -ntomp 24’, where grompp produces the .tpr file for the simulation to be run, utilizing the topology and gromacs file produced by INSANE. Finally, the simulation can be started using mdrun with the previously made .tpr file.

SI 2. GROMACS MDP File Parameters

The information below is the parameters used in each .mdp file for GROMACS. 0-min.mdp and 1-md.mdp are the minimization and equilibration for non-cholesterol containing membranes. 2-prod.mdp contains the parameters for the production run.

0-min.mdp:

```
integrator               = steep
nsteps                   = 10000
nstxout                  = 0
nstfout                  = 0
nstlog                   = 100

cutoff-scheme            = Verlet
nstlist                  = 20
pbc                      = xyz
verlet-buffer-tolerance  = 0.005

coulombtype              = reaction-field
rcoulomb                 = 1.1
epsilon_r                = 15    ; 2.5 (with polarizable water)
epsilon_rf               = 0
vdw_type                 = cutoff
vdw-modifier             = Potential-shift-verlet
rvdw                     = 1.1
```

1-md.mdp:

```
; TIMESTEP IN MARTINI
; Most simulations are numerically stable
; with dt=40 fs, some (especially rings and polarizable water) require 20-30 fs.
; Note that time steps of 40 fs and larger may create local heating or
; cooling in your system. Although the use of a heat bath will globally
; remove this effect, it is advised to check consistency of
; your results for somewhat smaller time steps in the range 20-30 fs.
; Time steps exceeding 40 fs should not be used; time steps smaller
; than 20 fs are also not required unless specifically stated in the itp file.

define                   = -DBILAYER_LIPIDHEAD_FC=10
integrator               = md
tinit                    = 0.0
dt                       = 0.020
nsteps                   = 1000000

nstxout                  = 0
nstvout                  = 0
nstfout                  = 0
nstlog                   = 10000 ; Output frequency for energies to log file
nstenergy                = 10000  ; Output frequency for energies to energy file
nstxout-compressed       = 10000  ; Output frequency for .xtc file
compressed-x-precision   = 100

cutoff-scheme            = Verlet
nstlist                  = 50
pbc                      = xyz
verlet-buffer-tolerance  = 0.005

epsilon_r                = 15 ; 2.5 (with polarizable water)
coulombtype              = reaction-field
rcoulomb                 = 1.1
vdw_type                 = cutoff ;(for use with Verlet-pairlist)
rvdw                     = 1.1 ;(for use with Verlet-pairlist)

tcoupl                   = v-rescale
tc-grps                  = MEMB SOLV
```

```
tau_t                    = 1.0  1.0
ref_t                    = 310 310

; Pressure coupling:
Pcoupl                   = C-rescale  ; parrinello-rahman
; Pcoupltype               = isotropic  ; semiisotropic
Pcoupltype               = semiisotropic
tau_p                    = 6.0       ; 12.0  ;parrinello-rahman is more stable with larger tau-p,
DdJ, 20130422
compressibility          = 4.5e-7 4.5e-7
ref_p                    = 1.0  1.0

gen_vel                  = yes
gen_temp                 = 310
gen_seed                 = 473529

constraints              = none
constraint_algorithm     = Lincs
continuation             = no
lincs_order              = 4
lincs_warnangle          = 30

refcoord_scaling         = all
```

2-prod.mdp:

```
integrator               = md
tinit                    = 0.0
dt                       = 0.020
nsteps                   = 50000000

nstxout                  = 50000
nstvout                  = 50000
nstfout                  = 50000
nstlog                   = 50000
nstenergy                = 50000
nstxout-compressed       = 50000
compressed-x-precision   = 100

cutoff-scheme            = Verlet
nstlist                  = 50
pbc                      = xyz
verlet-buffer-tolerance  = 0.005

epsilon_r                = 15
coulombtype              = reaction-field
rcoulomb                 = 1.1
vdw_type                 = cutoff
rvdw                     = 1.1
vdw-modifier             = Potential-shift-verlet

tcoupl                   = v-rescale
tc-grps                  = MEMB SOLV
tau_t                    = 1.0  1.0
ref_t                    = 310 310

; Pressure coupling:
Pcoupl                   = Parrinello-rahman
Pcoupltype               = semiisotropic
tau_p                    = 12.0
compressibility          = 4.5e-5 4.5e-5
ref_p                    = 1.0  1.0

; GENERATE VELOCITIES FOR STARTUP RUN:
```

```
gen_vel                   = no
refcoord_scaling          = all
```

SI 3. GROMACS Mean Square Displacement and Diffusion Constant Determination

To evaluate the lateral diffusion constant, the trajectories were first converted to remove the overall center of mass through use of the following command:

gmx trjconv -f {.xtc file} -n {index file} -s {.tpr file} -center -o {output.xtc}

following which, the mean-square displacement was computed through the next command:

gmx msd -f {.xtc file} -o {output.xvg} -n {index} -lateral z

of which the output file contains the time-labelled mean-square displacement data. This data can then have a linear fit applied to it, the slope of which will be directly proportional to the diffusion constant, by a factor of 2 times the dimensionality (2 for x-y lateral diffusion).

The area per lipid and membrane thickness analysis were performed based on the resulting trajectories using in-house Jupyter Lab python scripts utilizing MDAnalysis.

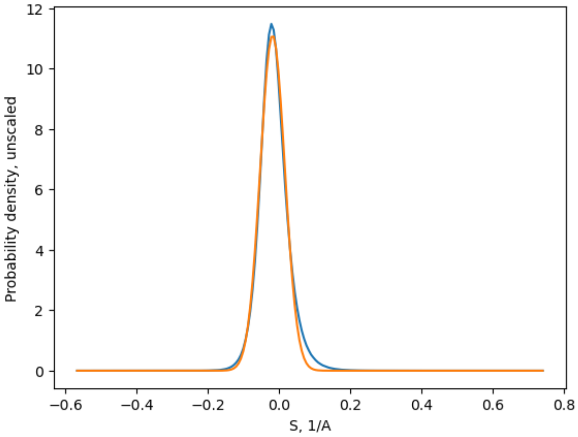

Figure S1. Quadratic fit of splay to probability density for RSF bending modulus.

Table S1. Replicability of experiments; Statistics of 5 trials of 100% POPC (in $k_B T$)

Analysis Method	Mean ± STD
q^{-4}	34.2 ± 0.4 (1.23%)
BW-DCF	24.8 ± 0.3 (1.15%)
RSF	24.3 ± 0.2 (0.719%)

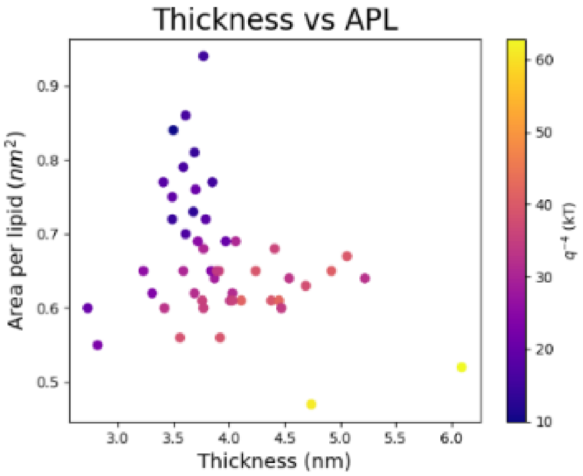

Figure S2. Color coded bending modulus of Thickness vs APL graph.

This plot shows that the high APL values greater than 0.7 nm^2 exclusively have bending moduli at the lower end of the spectrum, and as the APL approaches the more populated section between 0.6 and 0.7 nm^2, the bending modulus is more governed by the thickness of the membrane, but a key relationship has yet to be seen within these geometric parameters of the membranes constituents.

Figure S4. Coarse-grained lipids, tail groups and head groups

This section shows the coarse-grained structures of the lipids ran in simulation for this work.

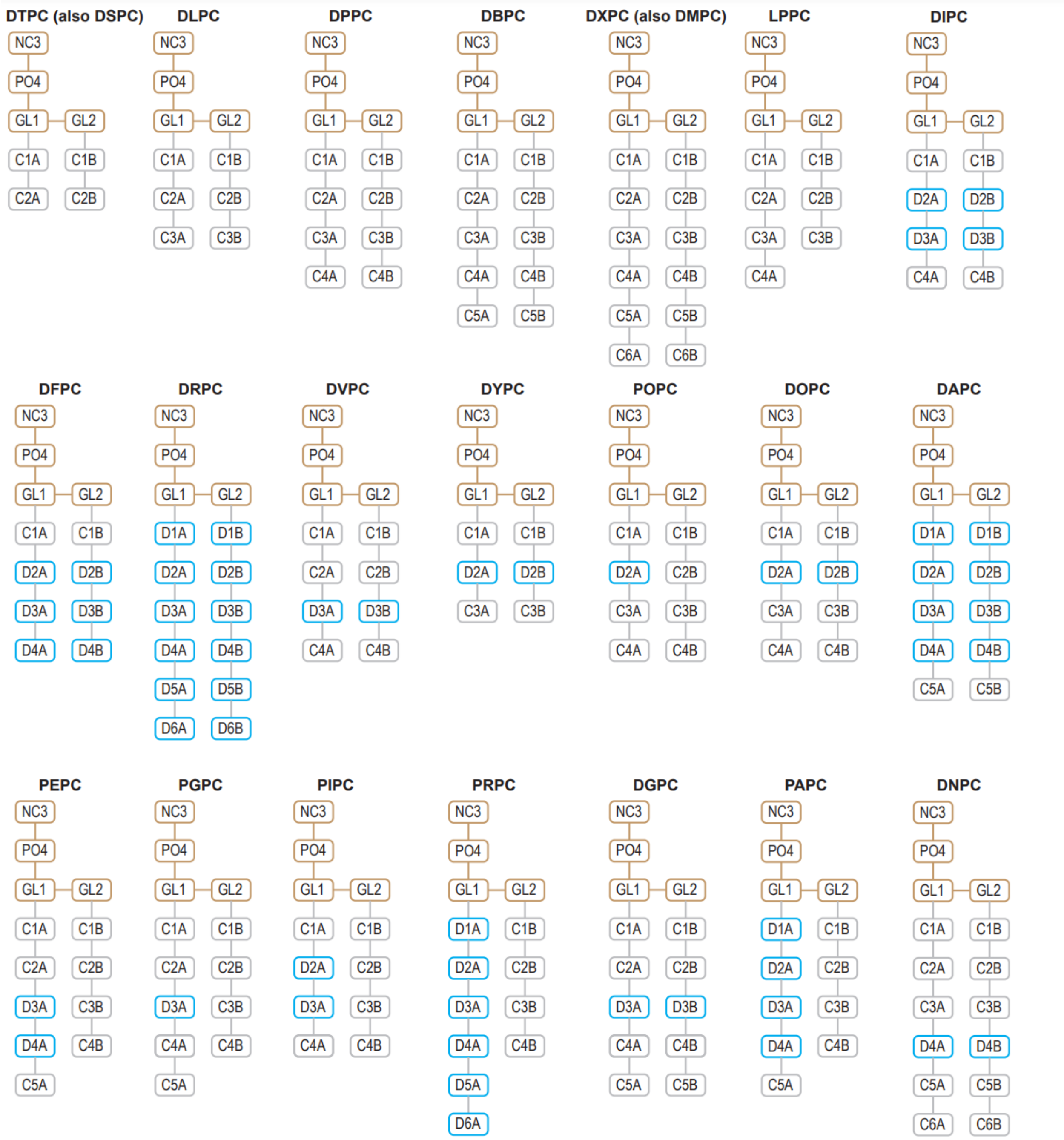

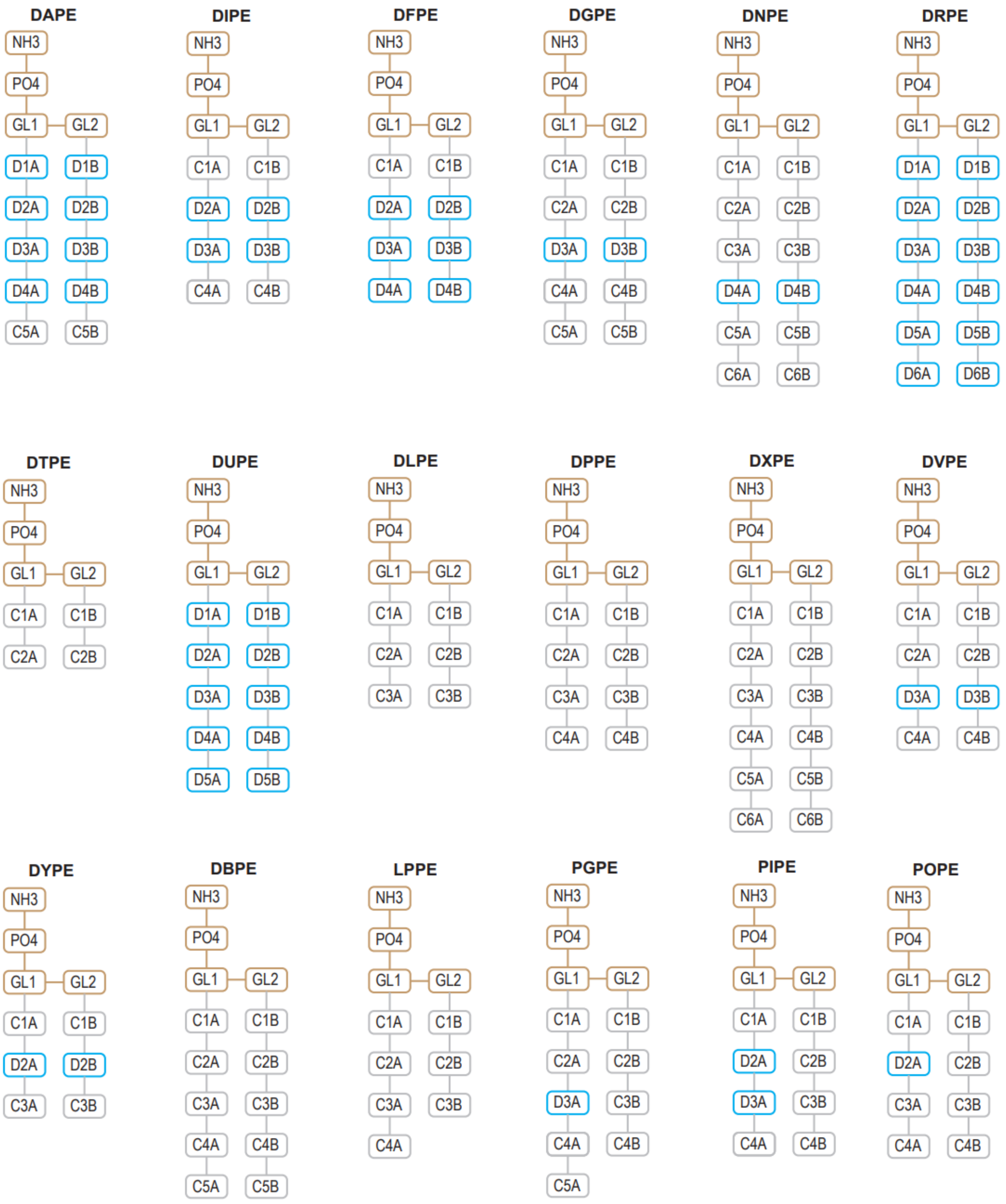
DAPE
NH3 PO4 GL1 GL2 D1A D1B D2A D2B D3A D3B D4A D4B C5A C5B
DIPE
NH3 PO4 GL1 GL2 C1A C1B D2A D2B D3A D3B C4A C4B
DFPE
NH3 PO4 GL1 GL2 C1A C1B D2A D2B D3A D3B D4A D4B
DGPE
NH3 PO4 GL1 GL2 C1A C1B C2A C2B D3A D3B C4A C4B C5A C5B
DNPE
NH3 PO4 GL1 GL2 C1A C1B C2A C2B C3A C3B D4A D4B C5A C5B C6A C6B
DRPE
NH3 PO4 GL1 GL2 D1A D1B D2A D2B D3A D3B D4A D4B D5A D5B D6A D6B
DTPE
NH3 PO4 GL1 GL2 C1A C1B C2A C2B
DUPE
NH3 PO4 GL1 GL2 D1A D1B D2A D2B D3A D3B D4A D4B D5A D5B
DLPE
NH3 PO4 GL1 GL2 C1A C1B C2A C2B C3A C3B
DPPE
NH3 PO4 GL1 GL2 C1A C1B C2A C2B C3A C3B C4A C4B
DXPE
NH3 PO4 GL1 GL2 C1A C1B C2A C2B C3A C3B C4A C4B C5A C5B C6A C6B
DVPE
NH3 PO4 GL1 GL2 C1A C1B C2A C2B D3A D3B C4A C4B
DYPE
NH3 PO4 GL1 GL2 C1A C1B D2A D2B C3A C3B
DBPE
NH3 PO4 GL1 GL2 C1A C1B C2A C2B C3A C3B C4A C4B C5A C5B
LPPE
NH3 PO4 GL1 GL2 C1A C1B C2A C2B C3A C3B C4A
PGPE
NH3 PO4 GL1 GL2 C1A C1B C2A C2B D3A C3B C4A C4B C5A
PIPE
NH3 PO4 GL1 GL2 C1A C1B D2A C2B D3A C3B C4A C4B
POPE
NH3 PO4 GL1 GL2 C1A C1B D2A C2B C3A C3B C4A C4B

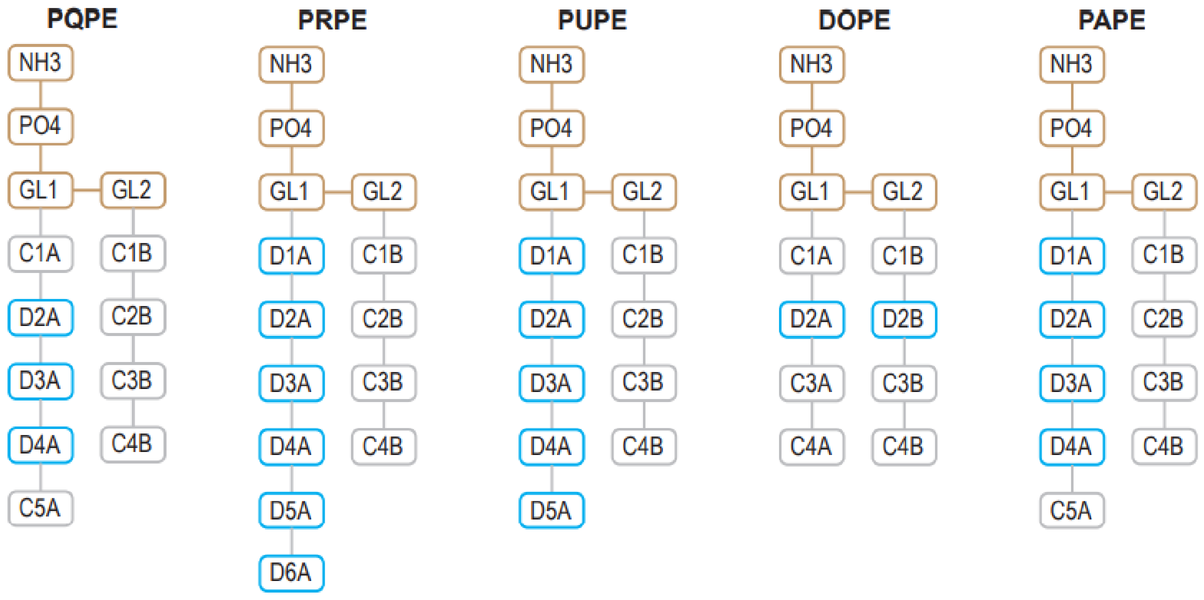
PQPE
NH3
PO4
GL1
GL2
C1A
C1B
D2A
C2B
D3A
C3B
D4A
C4B
C5A
PRPE
NH3
PO4
GL1
GL2
D1A
C1B
D2A
C2B
D3A
C3B
D4A
C4B
D5A
D6A
PUPE
NH3
PO4
GL1
GL2
D1A
C1B
D2A
C2B
D3A
C3B
D4A
C4B
D5A
DOPE
NH3
PO4
GL1
GL2
C1A
C1B
D2A
D2B
C3A
C3B
C4A
C4B
PAPE
NH3
PO4
GL1
GL2
D1A
C1B
D2A
C2B
D3A
C3B
D4A
C4B
C5A

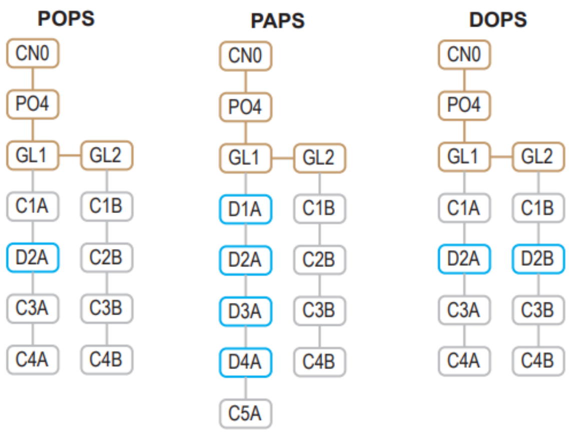

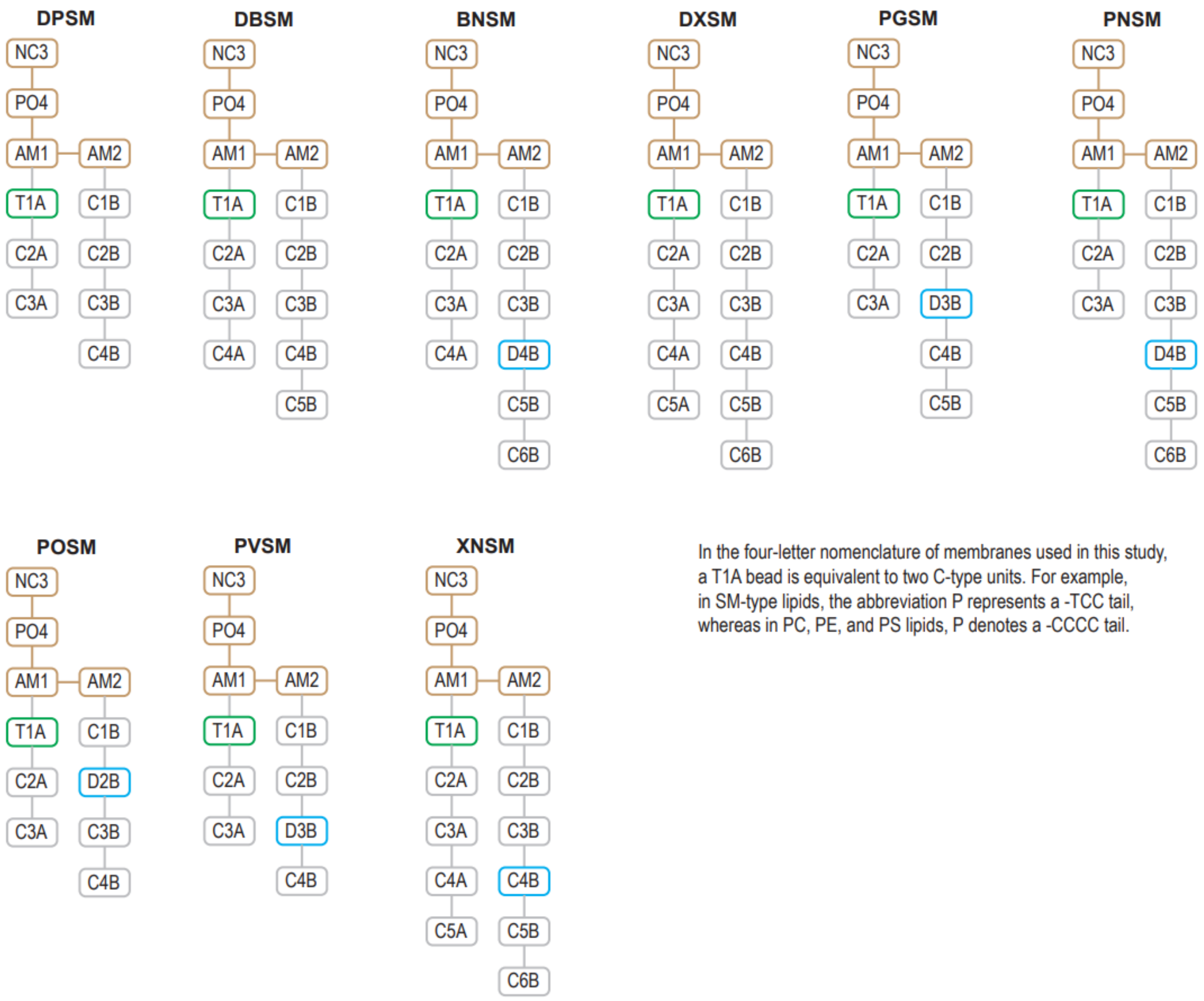

In the four-letter nomenclature of membranes used in this study, a T1A bead is equivalent to two C-type units. For example, in SM-type lipids, the abbreviation P represents a -TCC tail, whereas in PC, PE, and PS lipids, P denotes a -CCCC tail.

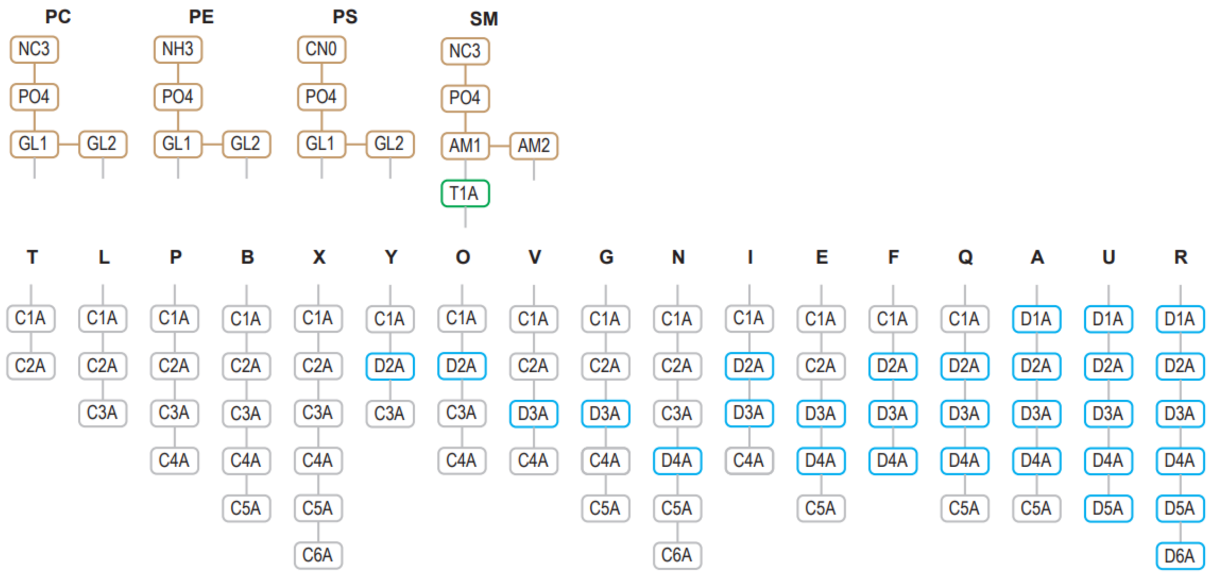

Table S2. Lipid geometrical properties, bending modulus and diffusion from Coarse-Grained Molecular Dynamics Simulations

Lipid type	APL (nm^2)	D_B (nm)	$\kappa(q^{-4})(k_BT)$	κ(BW-DCF) (k_BT)	κ(RSF) (k_BT)	D (10^{-6} cm^2/s)
100% DTPC	0.6	2.73	20.11	16.16	30.68	1.705
100% DLPC	0.6	3.42	32.8	24.25	31.02	1.079
100% DGPC	0.68	4.41	36.03	27.99	21.86	0.5956
100% DNPC	0.67	5.06	39.68	36.97	21.49	0.3503
100% PEPC	0.69	4.06	30.44	20.31	21.66	0.7977
100% DYPC	0.65	3.23	25.79	18.22	25.32	1.165
100% DVPC	0.65	3.91	36.57	25.8	24.78	0.7237
100% DFPC	0.77	3.41	17.81	12.48	18.08	1.489
100% LPPC	0.61	3.76	35.55	28.35	29.82	0.9979
100% PGPC	0.65	4.24	39.1	30.99	24.66	0.6036
100% DAPC	0.86	3.61	15.89	10.16	13.36	1.495
100% DTPE	0.55	2.82	22.91	15.69	40.58	1.308
100% DLPE	0.56	3.56	38.34	23.5	41.88	0.913
100% DOPE	0.64	3.87	28.7	14.48	26.12	0.7498
100% DIPE	0.7	3.61	16.56	9.47	20.2	1.251
100% DFPE	0.72	3.49	14.17	8.84	20.06	1.255
100% DGPE	0.64	4.54	33.16	19.36	25.51	0.4402
100% DAPE	0.81	3.69	13.72	7.13	15.78	1.41
100% DNPE	0.64	5.22	32.33	23.77	25.82	0.4245
100% PGPE	0.61	4.38	39.22	23.22	29.99	0.5107
100% PQPE	0.69	3.97	20.2	11.02	22.34	0.8613
100% DBSM	0.61	4.45	42.07	41.11	29.75	0.5308

100% PVSM	0.62	3.69	31.8	27.97	28.01	0.7873
100% BNSM	0.63	4.69	37.69	38.71	23.56	0.5073
100% PGSM	0.62	4.03	31.37	26.29	24.31	0.6973
100% PNSM	0.6	4.47	34.25	31.28	23.09	0.5302
100% POSM	0.65	3.59	30.21	24.44	25.03	0.8901
100% XNSM	0.65	4.92	40.72	41.22	24.87	0.4179
100% DIPC	0.75	3.49	19.05	12.99	17.96	1.292
100% DOPC	0.68	3.77	30.47	21.36	21.99	1.0919
100% DPPC	0.61	4.10	41.23	34.66	30.14	0.7735
100% DPSM	0.6	3.77	35.05	30.39	29.44	0.7399
100% DVPE	0.61	4.01	33.34	18.04	29.44	0.6407
100% DXPE	0.46	6.5	58.2	135.47	135.87	0.01084
100% DYPE	0.62	3.31	23.74	13.57	29.88	1.0468
100% LPPE	0.56	3.92	38.9	24.15	40.1	0.7206
100% PAPC	0.76	3.7	20.31	13.31	17.43	1.2687
100% PAPE	0.72	3.79	18.2	9.58	18.42	1.0177
100% PIPC	0.69	3.72	25.14	16.96	21.31	0.9053
100% PIPE	0.65	3.84	24.33	12.31	25.06	0.8508
100% POPC	0.65	3.89	34.27	25.84	24.57	0.7326
100% POPE	0.61	4.03	35.17	19.10	30.14	0.6933
100% PUPE	0.74	3.68	13.27	7.38	13.96	1.0165
100% DRPC	0.94	3.77	15.06	8.66	12.74	1.3079
100% DUPE	0.84	3.5	9.91	4.74	14.43	1.2569
100% PRPE	0.77	3.85	15.24	6.22	16.44	0.7569
100% PUPC	0.79	3.59	16.03	10.57	14.92	1.1821
100% DBPC	0.61	4.78	40.48	45.62	29.97	0.4883
100% DPPE	0.54	4.41	47.88	46.3	55.41	0.3708
100% DXPC	0.62	5.45	37.97	51.81	29.68	0.3381
100% DXPE	0.46	6.47	70.22	84.44	142.7	0.02195
100% DXSM	0.61	5.12	37.76	52.64	29.57	0.3488

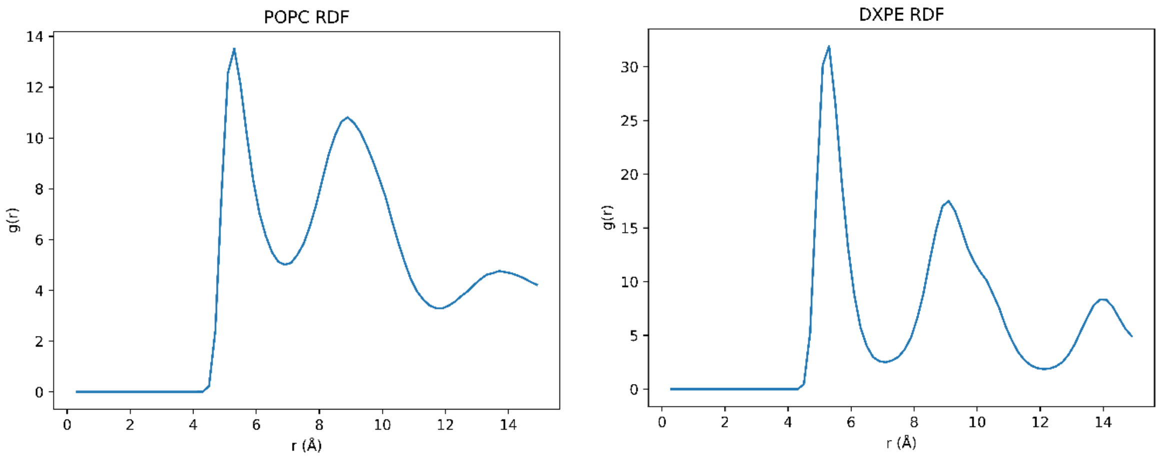

Figure S5. Radial distribution function of POPC and DXPE membranes. DXPE sharpened peaks indicate a more ordered (gel) phase.
